# Deep learning for cardiac CT segmentation for congenital heart disease: A systematic review

**DOI:** 10.1371/journal.pone.0357600

**Published:** 2026-09-29

**Authors:** Sinling Tiffany Yu, Zahra Amini, Shereen Fouad, Jan Novak, Antonio Fratini

**Affiliations:** 1 Engineering for Health Research Centre, Aston University, Aston Triangle, Birmingham, United Kingdom; 2 Aston Institute of Health & Neurodevelopment, Aston University, Aston Triangle, Birmingham, United Kingdom; 3 School of Computer Science and Digital Technologies, Aston University, Aston Triangle, Birmingham, United Kingdom; University of Salerno: Universita degli Studi di Salerno, ITALY

## Abstract

**Purpose:**

Deep Learning (DL) has transformed cardiac image segmentation, yet its application to congenital heart disease (CHD) remains underexplored, with no prior systematic review in this emerging domain. This review evaluates current DL approaches for CHD CT segmentation, identifies best performing model architectures, assesses quality of reporting and highlights challenges for clinical implementation.

**Materials and methods:**

Following PRISMA guidelines, six databases: PubMed, IEEE Xplore, MDPI, Science Direct, Web of Science, and Scopus were searched (2004–2025). Extracted characteristics include source of dataset, ground truth labelling approach, DL architecture and evaluation methods. Reporting quality was evaluated using the Checklist for Artificial Intelligence in Medical imaging (CLAIM).

**Results:**

14 studies met the inclusion criteria, reflecting the emergence of this research area. The UNet architecture and its variants dominated (8 studies). The highest reported Dice Similarity Coefficient (DSC) for cardiac structures segmentation was achieved utilising an advanced hybrid ResNet-based model (Aorta, DSC = 0.945). Well documented areas include the training approach (12 studies) and model description (all). Only two studies performed external validation, and two assessed inter- and intra-rater variability. No studies reported sample size determination and handing of missing data. Small vascular structures consistently underperformed compared to whole heart segmentation.

**Conclusion:**

Deep learning models show strong potential for CHD CT segmentation but remain limited by small datasets, inconsistent reporting and lack of clinical evaluation. Advancing this field requires multidisciplinary collaboration, standardised reporting, and integration of clinical co- design. As the first comprehensive review in this area, we provide a strong baseline for evaluating future studies.

## 1. Introduction

Congenital heart disease (CHD) is a chronic, life-long condition and the leading cause of mortality from birth defects worldwide [1]. It is defined as the structural abnormality of the heart or great vessels that occurs during foetal development. These morphological abnormalities lead to complications with cardiac function and circulation, often requiring early intervention and lifelong medical management [2]. Most CHD cases are diagnosed prenatally, through prenatal screening and foetal echocardiography; however, some children are born with undetected CHD and are only diagnosed postnatally. In critical cases where immediate intervention is necessary a prompt diagnosis is essential [3]. Surgical and interventional planning for CHD remains a challenge due to the small anatomy and unique nature of each malformation [4]. Hence, a personalised approach to diagnosis and surgical planning is essential for optimising patient outcomes.

To support this personalised approach, detailed imaging of cardiac anatomy is essential. CT imaging is usually the modality of choice for evaluating CHD due to its rapid acquisition time and ability to produce high-resolution images. Image segmentation can be employed to accurately identify regions of interests within these images, and traditionally, is performed manually by a clinician or radiologist, but this is time consuming and subject to inter and intra-user variability [5]. Semi-automated segmentation tools which require minimal user input use methods such as thresholding and region-growing, combined with imaging properties and clinical knowledge to delineate structures and features in the image [6]. Growing research into segmentation and 3D reconstruction has demonstrated successful use of 3D models for surgical planning, teaching, and communication purposes [7, 8].

Recent studies highlight significant backlogs and pressure amongst radiologists due to increasing demand for imaging services [9]. To address these challenges, decision support systems have been proposed covering a broad range of applications such as clinical documentation, diagnostic support and risk prediction [10, 11].

Central to many of these decision support systems is Artificial Intelligence (AI). AI based models have become increasingly prominent in the field of biomedical research and clinical practice [12]. One significant approach is deep learning (DL), a subset of machine learning (ML) that involves the use of neural networks with multiple layers to automatically extract features from large datasets. This enables DL to effectively model complex patterns and relationships in data, making it suitable for a variety of tasks such as image segmentation, disease detection, and predictive analytics [13, 14].

This systematic review aims to provide an in-depth overview of current research on deep learning techniques for cardiac CT segmentation in congenital heart disease. Previous systematic reviews have investigated deep learning methods for cardiac image segmentation across various modalities, including studies focusing on classification of heart sounds [15], echocardiograms [16], and cardiac MRIs [17, 18]. Alnasser et al (2024) provided a comprehensive systematic review on deep learning methods for cardiac structure segmentation of CT images [19]. However, this primarily addressed cardiovascular diseases (CVD) in adults, which often cover acquired conditions rather than congenital heart disease.

CHD presents unique challenges and differs significantly as it includes structural abnormalities present at birth, affecting cardiac function in ways that are distinct from adult-onset CVDs, and require specialised imaging and analysis techniques. Despite the importance of accurate cardiac CT segmentation for diagnosis and management of CHD, there is a lack of reviews focusing on this area. To the best of our knowledge, no review has specifically addressed deep learning techniques for cardiac CT segmentation in paediatric congenital heart disease. Therefore, we aim to address this gap through systematically reviewing current literature, summarising methodological trends, performance, and in addition provide a standardised reporting comparison of the selected studies using the most up-to-date version of the Checklist for Artificial Intelligence in Medical Imaging (CLAIM) [20]. Together, this provides a strong benchmark for current progress and guidance for further research and clinical translation.

## 2. Methods

A systematic review was conducted in accordance with the Preferred Reporting Information for Systematic reviews and Meta-Analysis (PRISMA) guidelines. This review was not prospectively registered on PROSPERO. The full review protocol, including predefined search strategies and eligibility criteria was developed prior to conducting the review. Further details can be found in S1 File in supplementary materials.

### 2.1. Study selection

A comprehensive search was conducted across six databases: PubMed, IEEE Xplore, MDPI, Science Direct, Web of Science, and Scopus including publications from 2004 to 2025. An example of the search string used for Web of Science is: ((ALL = Congenital heart disease) AND (ALL = CT) AND (ALL = Cardiac) AND (ALL = Segmentation) AND (ALL = Artificial Intelligence)) OR ((ALL = Congenital heart disease) AND (ALL = CT) AND (ALL = Cardiac) AND (ALL = Segmentation) AND (ALL = Machine learning)) OR ((ALL = Congenital heart disease) AND (ALL = CT) AND (ALL = Cardiac) AND (ALL = Segmentation) AND (ALL = Deep learning)). Full search strings and search strategy for all databases can be found in S1 File of supplementary materials. Endnote X9.3.3 was used to manage and screen references. Dual independent screening of the Title, Abstract and Full Text were performed by SY and AF, any disagreements were resolved through discussion and consensus. The last search was conducted 31^st^ October 2025.

This systematic review aims to answer the following questions:

- What type of DL models have been used for cardiac CT segmentation, and which models demonstrate better performance?
- How do current studies report study design and model performance?
- What are the current challenges and future research directions in integrating DL-based cardiac CT segmentation tools into clinical practice?

### 2.2. Eligibility criteria

Both paediatric and paediatric-adult mixed studies were eligible, provided that congenital heart disease cases were included and segmentation of cardiac specific anatomy was described. Studies were not limited by specific CT acquisition protocols to enable assessment of model generalisability. Segmentation was defined as a required inclusion criterion with studies that combined segmentation with downstream tasks such as classification included as long as the segmentation performance was explicitly reported. No limits on minimum reporting metrics were applied to enable a comprehensive assessment of reporting quality. The full inclusion and exclusion criteria are outlined in Table 1.

**Table 1:** Inclusion and exclusion criteria.

Inclusion criteria	Exclusion criteria
Studies published from 2004 to 2025	Studies with no mention of AI/ML/DL based methodology
Studies employing DL techniques for cardiac CT segmentation	Non-Journal papers, e.g.,: editorial, conferences, abstracts, surveys
Journal articles with novel approaches to cardiac CT segmentation for congenital heart disease	Studies not using CT imaging (e.g., Health records, Ultrasound, ECG)
Studies that investigate DL as a clinical tool for cardiac CT segmentation	
Studies that used datasets covering congenital heart disease	

### 2.3. Data extraction strategy

A detailed data extraction was conducted dual independently by the main investigator (SY) and secondary investigator (AF), with any discrepancies resolved by discussion and consensus. The following extracted information were documented into a table: general paper descriptions (authors, title, year, country), dataset characteristics (source of dataset, imaging type, dataset size, CHD pathology type, dataset split) and research design (model architecture, segmentation software, structures segmented, ground truth labelling approach). Study authors were not contacted regarding missing or unclear information as we wanted to accurately document reporting practices. In cases where methodological details were not reported in the publication or supplementary materials, this was documented as “Not mentioned”.

### 2.4. Reporting and quality assessment

Published literature often show variability and inconsistencies in documentation due to a lack of standardised reporting framework, making cross-study comparisons difficult. To address this, our systematic review uses the CLAIM 2024 checklist, a structured framework for documenting important aspects of AI studies, allowing for transparency, consistency, and reproducibility [20]. The final 14 studies were evaluated against 44 comprehensive criteria, covering aspects such as study design, dataset usage, data labelling, model training, evaluation methods and additional relevant factors. Five of the CLAIM criteria were excluded as they were deemed inapplicable or irrelevant for the purposes of comparison, resulting in 39 applicable items (Supplementary materials S2 File). CLAIM scoring was conducted dual independently by two investigators (SY and ZA), any discrepancies were resolved by discussion and consensus. Adherence to CLAIM was scored in a binary manner (Yes/No) by referring to the definitions and guidance provided. Overall compliance was calculated as (number of items scored Yes/ 39 applicable items) x 100%.

### 2.5 Risk of bias

Tools exist for assessing risk of bias in AI studies, but none are specifically designed for medical imaging segmentation tasks. QUADAS-2 developed in 2011 assesses diagnostic test accuracy whereas PROBAST+AI addresses prediction models [21, 22]. Deep learning segmentation models differ significantly as methodological bias is dependent on pixel and voxel level performance as well as annotation driven pipelines. Therefore, the PROBAST+AI framework was adapted to ensure the domain criteria’s address segmentation specific methodological considerations. Specifically, the ‘predictors’ domain which originally assessed clinical predictor variables, was reframed as ‘Input images and preprocessing’ to evaluate image acquisition and preprocessing pipelines, while the ‘Outcome’ domain was reframed as ‘Reference standard for segmentation’ to assess ground truth labelling, annotator qualifications, and inter-rater reliability. Signalling questions and performance metrics were also modified to reflect segmentation-specific considerations such as Dice Similarity Coefficient, Hausdorff distance and data leakage at the patient level. However, as this is a novel adaptation that has not been independently validated, the findings should be interpreted with this consideration in mind. The risk of bias assessment was performed dual independently by SY and ZA with disagreements resolved through discussion and consensus. The adapted template, including detailed instructions for implementation can be found in S3 File of supplementary materials.

## 3. Results

From the initial search, 1388 articles were identified. Following removal of duplicates, title, abstract and full-text screening, 13 articles were selected for review. Additionally, 1 article was identified from secondary sources, totalling 14 articles for the final systematic review (Fig 1).

**Fig 1:**
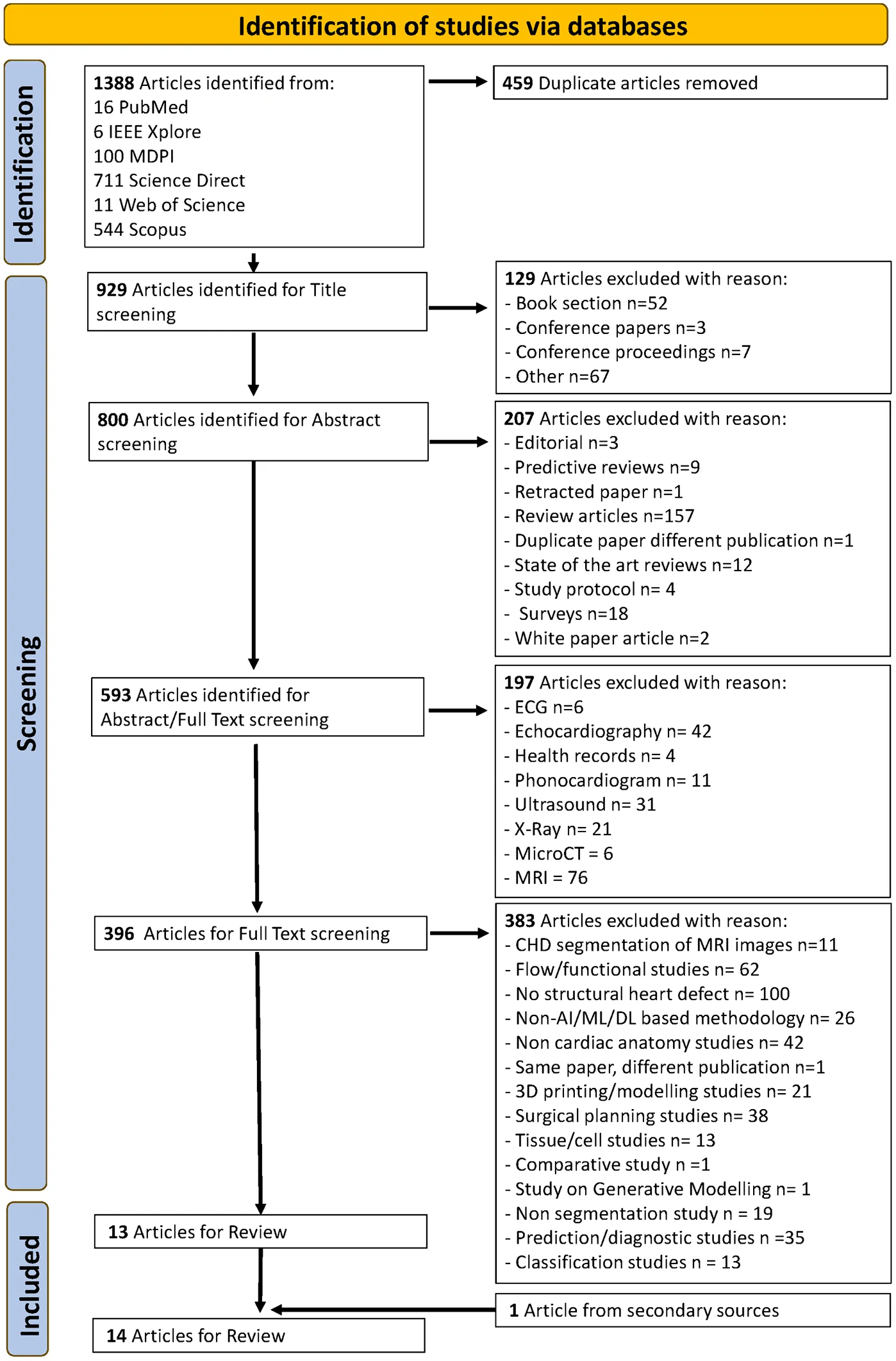
PRISMA flow diagram.

### 3.1. Publication and Geographical trends

Results show a diverse range of publication contributions from around the world with the highest number of publications originating from China (50%), followed by Korea and Japan (15%). The low number of publications per year highlights limited research in this area (Fig 2).

**Fig 2:**
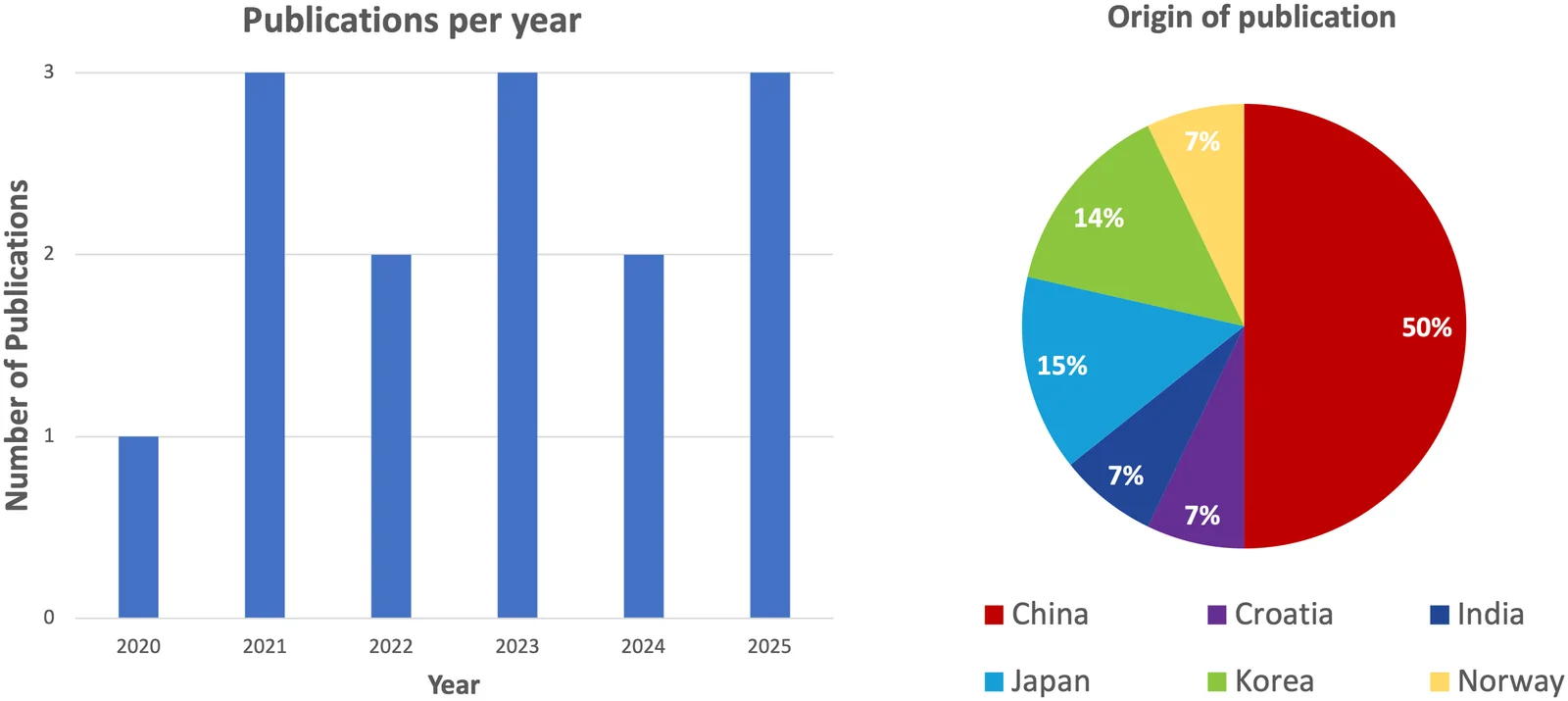
Plots showing origin of publication and number of publications per year.

### 3.2. Datasets

#### 3.2.1. Datasets characteristics.

The dataset size varied greatly between the 14 studies with the cohort size ranging from 20 to 3,750 participants. Ten studies used publicly available datasets [23–33] while the remaining conducted retrospective studies using data collected from their local hospitals [23,34–36]. Pei et al (2023) incorporated datasets from two different hospital institutions allowing for cross-institutional validation. Nainamalai et al (2022) used a publicly available dataset for model training and validated on a local hospital dataset. This approach enhanced the model’s reliability by enabling evaluation on real clinical data (Table 2). None of the articles provided an explanation on how the sample size was determined and only four of the selected studies provided information on patient demographics [23,24,35,36]. Commonly reported demographics include the age, gender, and types of congenital heart defect present (Table 2).

**Table 2:** Systematic review summary table.

Title	1st Author	Country of 1st Author	Publication Year	Imaging Type	CT Machine Type	Imaging Protocol	Source of Dataset	Demographics	Dataset Size (No. patients)	Train, Test, Validation split	Structures Segmented	CHD Pathology mentioned	Annotation software	Ground truth labeller	Ground truth validator
Anatomical attention-based prediction of postoperative pulmonary venous obstruction via CTA images	Pei, Yuchen [34]	China	2023	CTA	Not mentioned	Not mentioned	Local Hospital	Not mentioned	146	Not mentioned	LA, PV	TAPVC - Total anomalous pulmonary venous connection	ITKSnap	Cardiac Clinicians	Not mentioned
Binary segmentation of medical images using implicit spline representations and deep learning	Barrowclough, Oliver [29]	Norway	2021	CT	Not mentioned	Not mentioned	Public Open Source	Not mentioned	66	Training: 39, Testing: 14, Validation: 13	Ao, PA, LA, RA, LV, RV, Myo	ToF, TGA, Pulmonary artery sling, Anomalous drainage (AD), Common arterial trunk (CAT), Aortic arch anomalies(AAA), Single ventricle (SV), Pulmonary Atresia (PuA)	Not mentioned	Not mentioned	Not mentioned
Graph matching and deep neural networks based whole heart and great vessel segmentation in congenital heart disease	Yao, Zeyang [23]	China	2023	CTA	SOMATOM Definition Flash (Dual source CT)	Collimation: (96-128)x0.625mm, Rotation time:270ms, Slice thickness: 0.9mm, Reconstruction interval: 0.45mm, 40-50% RR interval	Public Open Source	Age: 1 months to 21 Years, 40% Female, 60% Male	68	Training: 51, Testing: 17	Ao, PA, LA, RA, LV, RV, Myo	Complex CHD types: ToF, TGA, Pulmonary artery sling (PAS), Anomalous pulmonary venous connection (APVC), Common arterial trunk (CAT), Aortic arch anomalies, Single ventricle (SV), Pulmonary atresia (PA)	Materialise Mimics	Radiologists	Not mentioned
Local integration of deep learning for advanced visualisation in congenital heart disease surgical planning	Nainamalai, Varatharajan [24]	Norway	2022	CTA	SOMATOM third-generation dual source CT	High pitch mode, contrast injected to upper extremities, two and three phase injection protocol (delay 18-26s, Ominipaque 6-30ml)	Both Public and Local	Local: Ages 1 day to 15 years	66	Training: 42, Testing: 12, Validation: 12	Whole heart (Ao+PA+LA+RA+LV+RV)	DORV, TGA, remote VSD, Single coronary, Sub pulmonary VSD, IAA, ccTGA, Mitral Valve hyplasia, Truncus arteriosus type 1&2, CoA.	3D Slicer	Cardiology Fellow (8 Yrs experience)	Paediatric Cardiologist (20 Yrs experience)
MMTLNET: Multi-modality transfer learning network with adversarial training for 3D whole heart segmentation	Liao, Xiangyun [25]	China	2020	CTA	Not mentioned	Not mentioned	Public Open Source	Not mentioned	120	Not mentioned	AA, PA, LA, RA, LV, RV, Myo	Not mentioned	Not mentioned	Not mentioned	Not mentioned
Whole heart segmentation using 3D FM-Pre-ResNet Encoder-Decoder based architecture with variational autoencoder regularization	Habijan, Marija [26]	Croatia	2021	CTA	64 slice CT scanner	Standard coronary CT angiography protocol	Public Open Source	Not mentioned	80	Training: 32, Validation: 8, Testing: 40	Ao, PA, LA, RA, LV, RV, LV-Myo	Not mentioned	Not mentioned	Clinical experts	Not mentioned
Automated heart segmentation using U-Net in pediatric cardiac CT	Yoshida, Akifumi [35]	Japan	2021	CTA	Not mentioned	Not mentioned	Local Hospital	15 Years and younger	20	Training: 19, Validation: 1	Whole heart (AA+LA+RA+LV+RV+PA, SVC, Myo, Intracardial adipose tissue)4 Chambers (LA+RA+LV+RV)	Not mentioned	Ziostation2	Researchers	Radiologist
A clinically applicable AI system for diagnosis of congenital heart diseases based on computed tomography images	Xu, Xiaowei [36]	China	2023	CT	Siemen’s SOMATOM Definition Flash (dual-source CT), Philip’s Brilliance iCT (256-slice CT), GE LightSpeed VCT (64-slice CT)	Not mentioned	Local Hospital	Median age: 4.45 Years, 40.6% female, 59.4% male	3750	Training: 1282, Testing: 2468	Ao, PA, LA, RA, LV, RV, Myo, SVC, IVC, PV	CHD (17 types) VSD, Single ventricle, ASD, Single atrium, ToF, DORV, TGA, Truncus arteriosus, PA, Interrupted AA, Ao Arch hypoplasia, CoA, Right AA, Anomalous pulmonary venous connection, PS, Double superior vena cava, PDA	3D Slicer	Resident physicians	Junior & Senior radiologists
A novel approach for multi-CHD prediction using IW-LNEF-DJRNN model based on 3D CT images	Anish, T.P [27]	India	2024	CT	Not mentioned	Not mentioned	Public Open Source	Not mentioned	110	Not mentioned	Ao, PA, LA, RA, LV, RV, Myo	16 CHD Types - AVSD, PA, CoA, TGA,DORV, Double AA, AA Hyplasia, DSVC, ASD,PDA, VSD, ToF, PA Sling, Common arterial trunk, APVD, Interrupted AA.	Not mentioned	Cardiovascular Radiologists	Not mentioned
Cardiac segmentation on CT images through shape-aware contour attentions	Park, Sanguk [28]	Korea	2022	CTA	Not mentioned	Not mentioned	Public Open Source	Not mentioned	60	Training & Validation: 20, Testing: 40	LA, PV	Not mentioned	Not mentioned	Trained students in Biomedical Engineering/Medical Physics	Observer with expertise in cardiac anatomy
A novel hybrid layer-based encoder-decoder framework for 3D segmentation in congenital heart disease	Zhu, Yaoxi [30]	China	2025	CT	Not mentioned	Not mentioned	Public Open Source	Not mentioned	110	Training: 88, Testing: 22	Ao, PA, LA, RA, LV, RV, Myo	ASD,AVSD, PDA, PuA, VSD, CA, ToF, TGA.	Not mentioned	Cardiovascular Radiologists	Not mentioned
Double-mix pseudo-label framework: enhancing semi-supervised segmentation on category-imbalanced CT volumes	Zhang, Luyang [31]	Japan	2025	CT	Not mentioned	Not mentioned	Public Open Source	Not mentioned	110	Training: 88, Validation: 11, Testing: 11	Ao, PA, LA, RA, LV, RV, Myo	Not mentioned	Not mentioned	Not mentioned	Not mentioned
Heart and great vessels segmentation in congenital heart disease via CNN and conditioned energy function postprocessing	Liu, Jiaxuan [32]	China	2024	CT	Not mentioned	Not mentioned	Public Open Source	Not mentioned	110	Training: 76, Validation: 7, Testing: 19	Ao, PA, LA, RA, LV, RV, Myo	16 CHD Types - AVSD, PA, CoA, TGA,DORV, Double AA, AA Hyplasia, DSVC, ASD,PDA, VSD, ToF, PA Sling, Common arterial trunk, APVD, Interrupted AA.	Not mentioned	Not mentioned	Not mentioned
Whole heart segmentation based on 3D contour-guided multi-head attention network from CT and MRI images	Li, Feiyan [33]	China	2025	CT	Not mentioned	Not mentioned	Public Open Source	Not mentioned	60	Training: 20, Testing: 40	Ao, PA, LA, RA, LV, RV, Myo	Not mentioned	Not mentioned	Not mentioned	Not mentioned

**Aorta(Ao), Ascending Aorta(AA), Pulmonary Artery(PA), Left Atrium(LA), Right Atrium(RA), Left Ventricle(LV), Right Ventricle(RV), Myocardium(Myo), Myocardium of the Left Ventricle (LV-Myo), Pulmonary Veins(PV), Superior Vena Cava(SVC), Inferior Vena Cava (IVC).

As with most clinical datasets, the ones used in the studies exhibited class imbalance, which can impact on model performance. For segmentation tasks, this imbalance can exist as disproportionate background regions to small foreground structures of interest. For classification tasks, imbalance often arises from unequal class distribution and representation of CHD types across the dataset (S1 Fig in supplementary materials documents the class imbalance present in classification tasks). Despite the potential influence of class imbalance on model training and evaluation, only two studies addressed or tried to mitigate this. In details: Nainamalai et al (2022) recognised the heterogeneity of the public and local datasets. Difference in model performances were assessed using a permutation test score with significant variation found between the distributions of the public and local test set. Moreover, Pei et al (2023) addressed severe class imbalance by implementing over sampling, duplicating the under-represented class. Additionally, a novel classification loss (Recall-MCAM Loss) was used, which encourages the model to focus on correctly identifying the underrepresented class [34].

#### 3.2.2. Imaging methods.

Within the selected studies, CT angiography was the most prevalent [23–26,28,34,35]. Imaging parameters varied between hospital institutions, with no standard protocol used across all (Table 2) – most studies using publicly available datasets did not mention key details on the imaging parameters. Xu et al (2023), investigated the use of multiple CT scanners to increase the generalisability of the results. Findings highlighted limited difference in performance loss (less than 2%) when the model was trained on data from one single machine and evaluated on others indicating good generalisation to new CT machines.

Image preprocessing strategies were reported in 13 studies [24–36], common techniques include cropping, resampling, intensity normalisation as well as down sampling. Anish et al (2024) utilised the Scaled Functionality-Centric Contrast Limited Adaptive Histogram Equalisation (SF-CLAHE), that enhances image contrast by adjusting pixel values based on the histogram of pixel intensities [27]. Zhu et al (2025) incorporated a double-mix augmentation module which blends labelled and pseudo labelled CT volumes by category using weight distributions [30]. (Table documenting pre-processing strategies in S1 Table).

### 3.3. Segmentation and labelling

#### 3.3.1. Investigated CHD types.

CHD conditions covered across the studies varied. Xu et al (2023) included the highest number of CHD types, classifying 17 types of CHD. Anish et al (2024) included 16 different types of CHD in their study while both Yao et al (2023) and Barrowclough et al (2021) explored 14 types of CHD. 10 types of CHD were mentioned in the dataset Nainamalai et al (2022) used. The full list of CHD types mentioned across the papers is summarised in (Table 2*).* Table 3 shows the dataset characteristics for the three open-source datasets mentioned across the 14 studies, with ImageCHD being most used.

**Table 3:** Dataset characteristics for open-source datasets mentioned across the 14 studies.

Dataset Name	Type	Year	Modality	Number of subjects	Labelled structures	Included pathologies
MM-WHS [49]	Open source/ Public	2017	CT/CTA	60	LV, RV, LA, RA, Myo, AO, PA	Myocardium infarction, Atrial fibrillation, Tricuspid regurgitation, Aortic valve stenosis, Alagille syndrome, Williams syndrome, Dilated cardiomyopathy, Aortic coarctation, Tetralogy of Fallot
CHD_68 [50]	Open source/ Public	2019	CT	68	LV, RV, LA, RA, Myo, AO, PA	Atrial septal defect, Ventricular septal defect, Atrioventricular septal defect, Anomalous drainage, Tetralogy of Fallot, Pulmonary artery sling, Patent ductus arteriosus, Coarctation, Common arterial trunk, Pulmonary stenosis, Aortic arch anomalies, Transposition of great arteries, Single ventricle, Pulmonary atresia
ImageCHD [51]	Open source/ Public	2020	CT	110	LV, RV, LA, RA, Myo, AO, PA	Atrial septal defect, Ventricular septal defect, Atrioventricular septal defect, Tetralogy of Fallot, Transposition of Great Arteries, Double outlet right Ventricle, Common arterial trunk, Coarctation, Aortic arch hypoplasia, Double aortic arch, Interrupted aortic arch, Pulmonary atresia, Anomalous pulmonary venous drainage, Double superior vena cava, Patent ductus arteriosus, Pulmonary artery sling

The ground truth labelling was reported in all studies. Source of ground truth labeller was described in 9 studies [23,24,26–30,34–36] as well as annotation tools used disclosed only across 5 studies, including: ITKSnap [34], 3D Slicer [24,36], Materialise Mimics [23], and Ziostation2 [35]. Two studies outlined strategies to minimise inter and intra variability during the ground truth labelling process [23,36]. The study by Xu et al (2023) implemented a multi-tiered approach; 17 resident physicians performed the initial segmentation, reviewed by 3 junior radiologists and any concerns further reviewed by a senior radiologist. In comparison, Nainamalai et al (2022) took on a 2-step approach, a cardiology fellow with 8 years of experience performed the segmentation which was then validated and approved by a paediatric cardiologist with 20 years of experience. Neither of the studies completed any quantitative assessments to evaluate variation between annotators.

### 3.4. Model characteristics, frameworks, and architectures

Two types of model frameworks were present across the studies: single -task and multi-task. Single-task frameworks focus on solving one specific problem such as segmentation whereas multi-task framework models are developed for more than one task (i.e., segmentation and classification). The full table documenting types of model frameworks across the studies can be found in S2 Table of the supplementary materials. Five main model architectures were identified across the 14 studies: UNet, CNN, RNN, GAN and Advanced hybrid-based models (*Supplementary materials* S3 Table). Table 4 provides a summary of the deep learning methods across the studies.

**Table 4:** Summary of deep learning methods for cardiac CT segmentation.

Application	Study	Imaging	Source of Dataset	Architecture Type	Description	Structure(s)
Multi-structure segmentation	Anish et al. (2024)	CT	Public open source	RNN-based	IWLNEF-DJRNN - Inverse weight with Logish Normalised Exponential Function-based Deep Jordan Recurrent Neural Network	Ao, PA, LA, RA, LV, RV, Myo
	Liao et al. (2020)	CTA	Public open source	Advanced hybrid	MMLNET - Multi-modality transfer learning network composed of GAN image generator, CNN discriminator and UNet segmentation units.	AA, PA, LA, RA, LV, RV, Myo
	Pei et al. (2023)	CTA	Local - 2 Hospitals	Advanced hybrid	UNet segmentation, CNN Classification, Features aggregation map (FAM) and Class activation map (CAM)	LA, PV
	Habijan et al. (2021)	CTA	Public open source	Advanced hybrid	3D FM-Pre-ResNet - Feature merge units guided with variational autoencoders (VAE)	Ao, PA, LA, RA, LV, RV, LV-Myo
	Zhu et al. (2025)	CT	Public open source	Advanced hybrid	Hybrid Layer-based Swin transformer framework with global volume mixing and local multihead attention	Ao, PA, LA, RA, LV, RV, Myo
	Li et al. (2025)	CT	Public open source	Advanced hybrid	Hybrid model combining 3D convolutional neural networks with Transformer attention mechanisms within a two stage UNet like architecture	Ao, PA, LA, RA, LV, RV, Myo
	Barrowclough et al. (2021)	CT	Public open source	UNet-based	UNet Implicit with adaptive average pooling	Ao, PA, LA, RA, LV, RV, Myo
	Yao et al. (2023)	CTA	Public open source	UNet-based	2D & 3D UNet for segmentation, connections extraction and graph matching for vessel classification	Ao, PA, LA, RA, LV, RV, Myo
	Liu et al. (2024)	CT	Public open source	UNet-based	Two-stage UNet based model with gated self-attention + conditioned energy function tailored for vessel segmentation	Ao, PA, LA, RA, LV, RV, Myo
	Xu et al. (2023)	CT	Local Hospital	UNet-based	Multi-task framework - ROI extraction, 2D & 3D UNet segmentation, domain knowledge based automatic diagnosis	Ao, PA, LA, RA, LV, RV, Myo, SVC, IVC, PV
	Zhang et al. (2025)	CT	Public open source	CNN-based	Semi-supervised Double-Mix framework with confidence-difficulty weights and cross-pseudo-supervision (CPS) co-training	Ao, PA, LA, RA, LV, RV, Myo
	Park et al. (2022)	CTA	Public open source	CNN-based	CDA-Net - Contour and distance transform guided attention network	AA, PA, LA, RA, LV, RV, LV-Myo
Whole heart segmentation	Yoshida et al. (2021)	CTA	Local Hospital	UNet-based	UNet for whole heart and 4 chamber segmentation	Whole heart (AA+LA+RA+LV+RV+PA, SVC, Myo, Intracardial adipose tissue) 4 Chambers (LA+RA+LV+RV)
	Nainamalai et al. (2022)	CTA	Public open source Local Hospital (Validation)	CNN-based	DenseVNet 3D CNN for Whole heart segmentation	Whole heart (Ao+PA+LA+RA+LV+RV)

**Aorta(Ao), Ascending Aorta(AA), Pulmonary Artery(PA), Left Atrium(LA), Right Atrium(RA), Left Ventricle(LV), Right Ventricle(RV), Myocardium(Myo), Myocardium of the Left Ventricle (LV-Myo), Pulmonary Veins(PV), Superior Vena Cava(SVC), Inferior Vena Cava (IVC)

#### 3.4.1. UNet variants.

The UNet is a popular model for biomedical image segmentation, it consists of a specialised encoder-decoder structure allowing the capture of high-resolution features as well as precise localisation [37]. Eight studies implemented UNet variants to enhance segmentation tasks [23,25,29,32–36]. Yoshida et al (2021) implemented a two-stage approach, first was segmentation of the whole heart followed by a refined four-chamber segmentation. Barrowclough et al (2021) introduced a UNet implicit architecture using implicit spline functions to model the segmentation boundary, this significantly reduced the computational complexity and memory use with results outperforming traditional pixel-based prediction CNNs. Yao et al (2023) proposed a 2-stage framework, integrating clinical knowledge, findings documented improved segmentation accuracy. Xu et al (2023) further enhanced segmentation with a multi-task framework which mirrored clinical workflows, this achieved accuracy comparable to junior radiologists and reduced diagnosis time significantly. Liu et al (2024) used a UNet with gated self-attention mechanisms and a novel conditioned energy function module specifically designed for great vessel refinement, this saw a 1.05–1.42% improvement in DSC, IOU and HD95.

#### 3.4.2. CNN variants.

Convolutional Neural Networks (CNNs) are among the most popular architectures, known for their ability to capture hierarchical features. Park et al (2022) introduced a CNN-based contour and distance transform-guided network, the network enhances segmentation accuracy by focusing on boundary regions. Nainamalai et al (2022) used a DenseVNet to reduce manual segmentation time. Zhang et al (2025) used a semi-supervised double-mix framework with confidence-difficulty weights and cross-pseudo-supervision (CPS) co-training, this demonstrated that even with 5% labelled data, meaningful segmentation could still be achieved.

#### 3.4.3. RNN variants.

Recurrent Neural Networks (RNNs), designed for pattern recognition in sequential data was used by Anish et al (2024). A multi-task framework for segmentation and classification was proposed, this incorporated clustering and feature selection to optimise segmentation, followed by an inverse weight with logish normalised exponential function-based deep Jordan recurrent neural network (IW-LNEF-DJRNN) to classify 16 types of congenital heart disease.

#### 3.4.4. GAN variants.

Liao et al (2020) proposed a multi-modality transfer learning network (MMLNET) to improve MRI to CT segmentation. The advanced hybrid structure was composed of three main units: a GAN based generator to transform MRI images to CT like images, a CNN-based discriminator unit to differentiate between synthetic and real data and a UNet based segmentation unit. Findings demonstrated superior performance compared to traditional CNN and UNet-based models.

#### 3.4.5. Advanced hybrid deep learning models.

Pei et al (2023) adopted a multi-task learning framework, the hybrid model combined UNet-based segmentation with a CNN classification model to predict postoperative pulmonary venous obstruction (PPVO), improved outcomes were achieved over single-task approaches. Habijan et al (2021) developed a hybrid model which combined a ResNet-based encoder for deep feature extraction with a Variational Autoencoder (VAE) regularisation to capture latent representatives. The UNet inspired encoder-decoder structure with skip connections achieved DSC values comparable to state-of-the-art (SOTA) models. Zhu et al (2025) introduced a novel hybrid layer-based encoder-decoder framework based on Swin transformer elements. The approach integrates global volume mixing modules (Global contextual mixer) with local volume based multi-head attention mechanisms (Local contextual self-attention), findings showed increased DSC values compared to SOTA models. Li et al. (2025) introduced a two-stage hybrid segmentation framework. This combined a 3D UNet based architecture with transformer style multi-head attention and contour-aware feature guidance. The model achieved higher DSC and improved boundary accuracy compared to five other SOTA models.

### 3.5. Evaluation and performance metrics

Segmentation performance was evaluated using pixel-based and overlap-based metrics (Fig 3). Pixel-based metrics, which include Precision, Recall, Accuracy and Sensitivity assesses the performance at pixel level. Overlap-based metrics including the Dice Similarity Coefficient and Jaccard Index measures the agreement between the model prediction and the ground truth segmentation. Sensitivity was the most used pixel-based metric, and DSC was the most used overlap-based metric. Only a small proportion of studies tested their model on an external dataset to check its generalisability to unseen data [24,34]. Similarly, only 8 studies performed robustness and sensitivity analysis to assess performance on conditions such as the presence of outliers, noise, and artifacts in the dataset [25,28,30–34,36].

**Fig 3:**
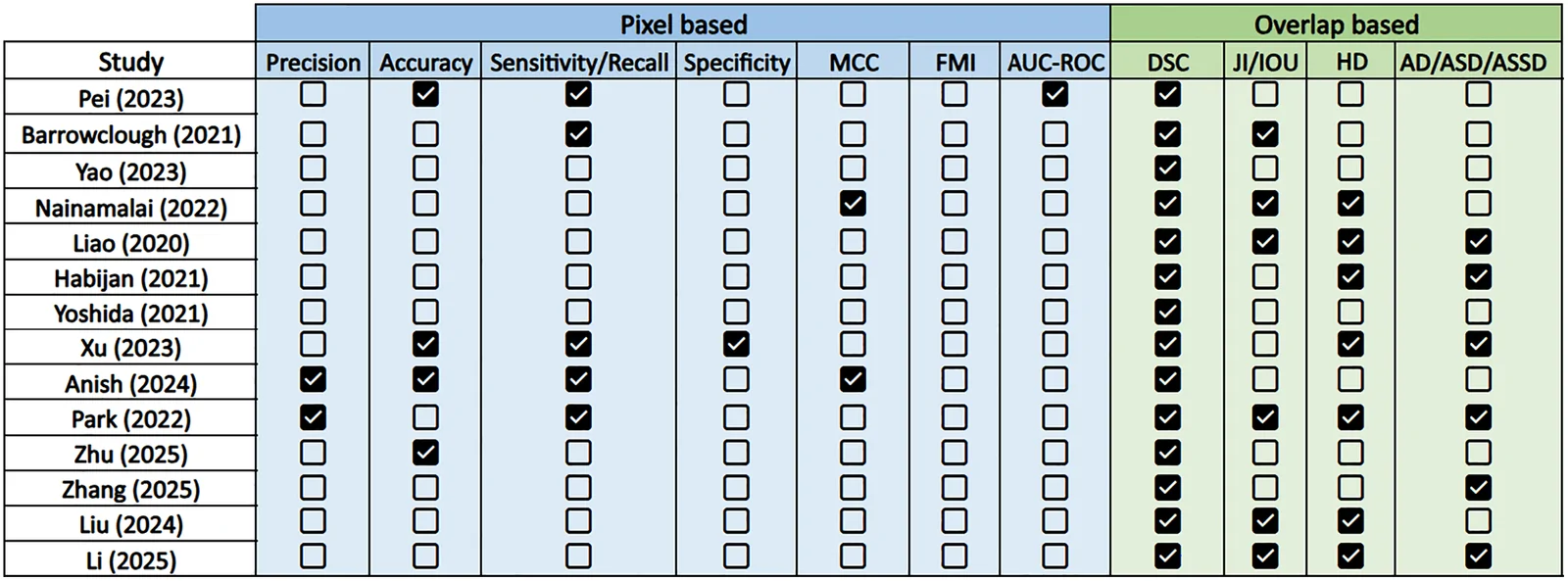
Performance metrics utilised across the studies (Matthews Correlation Coefficient (MCC), Fowlkes-Mallows Index (FMI), Area Under Curve (AUC), Receiver Operating Curve (ROC), Dice Similarity Coefficient (DSC), Jaccard Index (JI), Intersection Over Union (IOU), Average Distance (AD), Average Surface Distance (ASD), Average Symmetric Surface Distance (ASSD).

### 3.6. Segmentation performance

The DSC across the 14 studies varied between the structures segmented (Fig 4). Due to substantial heterogeneity across the studies including datasets, structures segmented and validation strategies, it is important to note that direct quantitative comparison of segmentation performance between models is not appropriate. The following reported performance metrics are summarised descriptively and should be interpreted within the context of each studies experimental setting. Two main approaches were taken, whole heart segmentation (which includes the entire blood pool of the four chambers and great vessels but without differentiations) and multi-structure segmentation (which distinguishes heart structures). Whole heart segmentation generally achieved higher reported DSC (value reaching 0.967 in one study), this is likely due to clearer anatomical boundaries. In contrast, smaller vessel structures such as the pulmonary veins (0.631), pulmonary arteries (0.671), SVC (0.521) and IVC (0.513) showcased a wider variability and range in reported DSC across studies, likely due to thinner walls and variable morphology making them harder to delineate, highlighting the need for improved training strategies. Habijan et al (2021) using a hybrid ResNet-based model, reported higher DSC for two structures (LA, Ao), however these results were obtained using a different dataset and validation strategies so should not be interpreted as directly outperforming other methods included in this review. To avoid misleading cross study comparisons, the complete table of quantitative results can be found in supplementary materials S4 Table and S2 Fig, where performance is interpreted based on task description.

**Fig 4:**
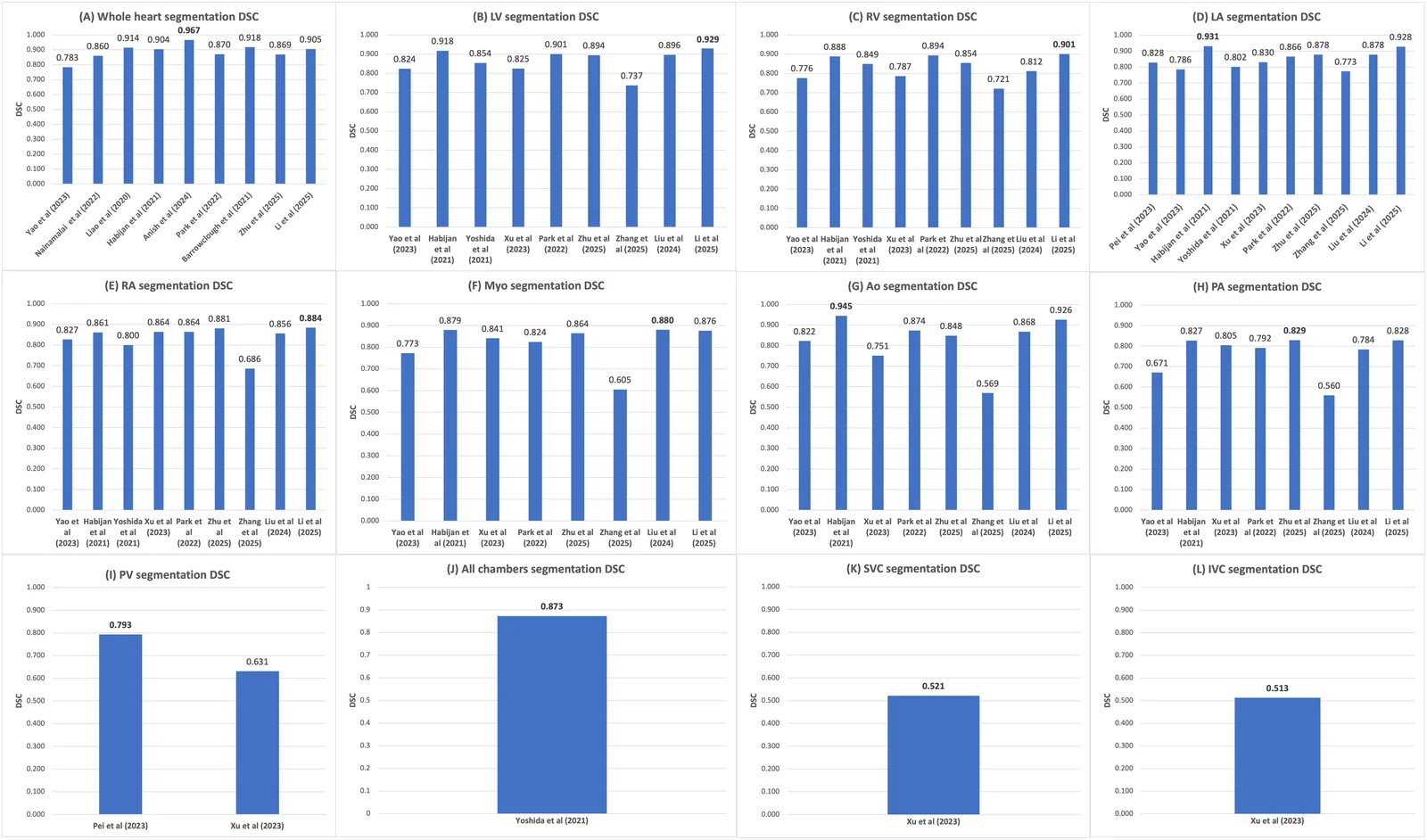
DSC for segmentation of different cardiac structures across 14 studies. Left Ventricle (LV), Right Ventricle (RV), Left Atrium (LA), Right Atrium (RA), Myocardium (Myo), Aorta (Ao), Pulmonary Artery (PA), Pulmonary Veins (PV), Superior Vena Cava (SVC), Inferior Vena Cava (IVC).

### 3.7. Risk of bias

Table 5 shows the risk of bias assessment for the 14 included studies based on the adapted PROBAST+AI framework. Model development and model evaluation were assessed separately using this framework. For model development, all studies were rated as unclear, mainly due to insufficient reporting of reference standard generation and analysis procedures. Domain specific assessment found ‘input images and preprocessing’ as the strongest domain with 12/14 studies (85.7%) reported as low risk [24–31,33–35,38]. The ‘reference standard for segmentation’ and ‘analysis’ domains showed greatest methodological concern with 11/14 studies (78.6%) reported as unclear [25–35]. Due to most studies utilising open-source datasets, key concerns include the lack of transparent annotation protocols, demonstration of consistent reference standard labelling and evidence of sample size adequate for model complexity.

**Table 5:** Risk of bias assessment using adapted PROBAST+AI.

Study	Risk of Bias according to modified PROBAST+AI Domain				
	Participants and Data Sources	Predictors - Input images and preprocessing	Outcomes - Reference standard for segmentation	Analysis	Overall
	Model Development				
**Pei et al (2023)**	Low	Low	Unclear	Low	**Unclear**
**Barrowclough et al (2021)**	Unclear	Low	Unclear	Unclear	**Unclear**
**Yao et al (2023)**	Low	Unclear	Low	Unclear	**Unclear**
**Nainamalai et al (2022)**	Low	Low	Low	Unclear	**Unclear**
**Liao et al (2020)**	Unclear	Low	Unclear	Low	**Unclear**
**Habijan et al (2021)**	Unclear	Low	Unclear	Unclear	**Unclear**
**Yoshida et al (2021)**	Low	Low	Unclear	Unclear	**Unclear**
**Xu et al (2023)**	Low	Unclear	Low	Low	**Unclear**
**Anish et al (2024)**	Unclear	Low	Unclear	Unclear	**Unclear**
**Park et al (2022)**	Unclear	Low	Unclear	Unclear	**Unclear**
**Zhu et al (2025)**	Low	Low	Unclear	Unclear	**Unclear**
**Zhang et al (2025)**	Unclear	Low	Unclear	Unclear	**Unclear**
**Liu et al (2024)**	Unclear	Low	Unclear	Unclear	**Unclear**
**Li et al (2025)**	Unclear	Low	Unclear	Unclear	**Unclear**
	**Model Evaluation**				
**Pei et al (2023)**	Low	Low	Low	Low	**Low**
**Barrowclough et al (2021)**	Unclear	Low	Unclear	Unclear	**Unclear**
**Yao et al (2023)**	Low	Low	Low	Low	**Low**
**Nainamalai et al (2022)**	Low	Low	Low	Unclear	**Unclear**
**Liao et al (2020)**	Low	Unclear	Low	High	**High**
**Habijan et al (2021)**	Low	Unclear	Low	Unclear	**Unclear**
**Yoshida et al (2021)**	Low	Low	Low	High	**High**
**Xu et al (2023)**	Low	Low	Low	Low	**Low**
**Anish et al (2024)**	Low	Low	Low	Unclear	**Unclear**
**Park et al (2022)**	Low	Unclear	Low	Unclear	**Unclear**
**Zhu et al (2025)**	Low	Low	Low	Unclear	**Unclear**
**Zhang et al (2025)**	Low	Low	Low	Unclear	**Unclear**
**Liu et al (2024)**	Low	Low	Low	Unclear	**Unclear**
**Li et al (2025)**	Low	Low	Low	Unclear	**Unclear**

Model evaluation demonstrated better methodological quality overall. Generally, low risk was reported across three domains (‘Participants and data sources’, ‘Input images and preprocessing’ and ‘reference standard for segmentation’). Three studies were reported low risk overall [23,34,36], two were reported high risk [25,35] while nine were reported unclear [24,26–33,38].

## 4. Discussion

Research on AI and deep learning applications in cardiology has predominantly focused on adult populations, with paediatric CHD, especially segmentation methods using cardiac CT significantly underexplored [39]. This systematic review addresses this research gap by specifically evaluating deep learning approaches for CHD segmentation in paediatric cardiac CT, an emerging field evident by the limited number of studies meeting the inclusion criteria and all the publications being within the past five years.

Across the 14 studies, several recurring strengths and gaps were identified that shape the current state of the field. Strengths include robust technical model performance, with UNet variants and advanced hybrid architectures achieving high segmentation performance for larger cardiac structures, alongside strong CLAIM compliance in domains such as model description, training approach, and performance metrics reporting. However, key gaps emerged in four areas: limited clinical evaluation and co-design with end users, inconsistent ground truth labelling practices, methodological and reporting limitations (particularly around sample size determination, handling of missing data, and terminology use), and underdeveloped explainability and interpretability strategies. These gaps are discussed in the following subsections.

### 4.1. Clinical evaluation and co-design

Whilst technical model performance is essential, clinical utility of deep learning models requires both robust clinician evaluation and meaningful co-design with healthcare professionals. Only two studies included clinician evaluation [23,36], highlighting a persistent bias towards quantitative performance metrics over qualitative feedback from clinicians. This imbalance limits the understanding of DL model usability, integration into clinical workflows and interpretability of outputs for end users. Without clinician input, key factors such as trust, and decision-making support remain unexplored [40]. Although clinicians, radiologists, and medical professionals are often identified as the end users of these tools their involvement in many studies often stops following the ground truth labelling stage. Only two studies integrated clinical domain knowledge into their model design [23,36]. Future research requires adopting a balanced approach combining quantitative performance metrics with qualitative clinician feedback as well as strong clinical engagement through multidisciplinary collaboration ensuring that the outputs are meaningful and align with clinical needs, increasing the likelihood of clinical integration [41,42].

### 4.2. Ground truth labelling

The ground truth is considered the ‘Gold Standard’, and it is a significant factor influencing the model’s prediction [43]. Despite its importance, over half of the studies did not mention the annotation tool used [25–33] and five of them did not disclose their ground truth labelling source [25,29,31–33]. In addition, across AI and deep learning publications, there is limited research on validating ground truth, addressing labelling inter and intra variability and establishing standard protocols, such as using medically certified software. The term ‘expert’ is also subjective; with some studies using trained students followed by validation by a clinician due to the impracticality of having clinicians manually annotating large datasets. With varying levels of expertise, approaches such as consensus through majority voting might also include biases.

### 4.3. Methodology and reporting

Our analyses revealed strong compliance to the CLAIM checklist (Fig 5, 6) across seven domains: use of AI, study aims, study goals, data sources, model description, performance metrics and internal evaluations. However, key areas require improvement. Notably, none of the reviewed studies mentioned how the intended sample size was determined (e.g., using power calculations) or how missing data was handled. Despite this being a commonly reported issue in DL studies, with the typical approach being that more data is better, this very much limits the rigour of the studies [44]. Model descriptions and training approaches were generally thorough providing clear documentation of software libraries, frameworks, training epochs and loss functions, the rationale for selecting hyperparameters was inadequately justified. With many reasons based on prior literature rather than experimental validation.

**Fig 5:**
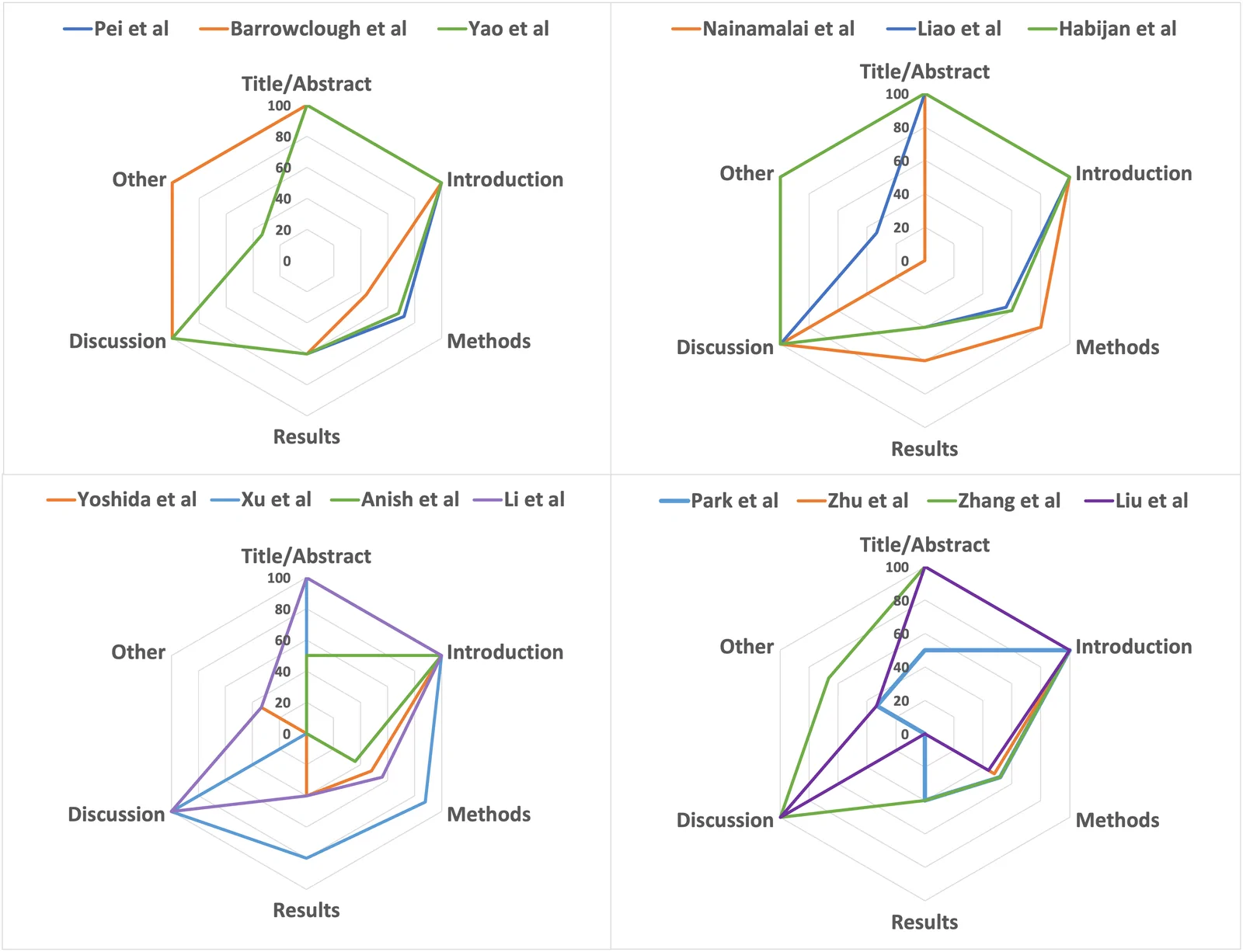
Compliance with the CLAIM checklist across six domains.

**Fig 6:**
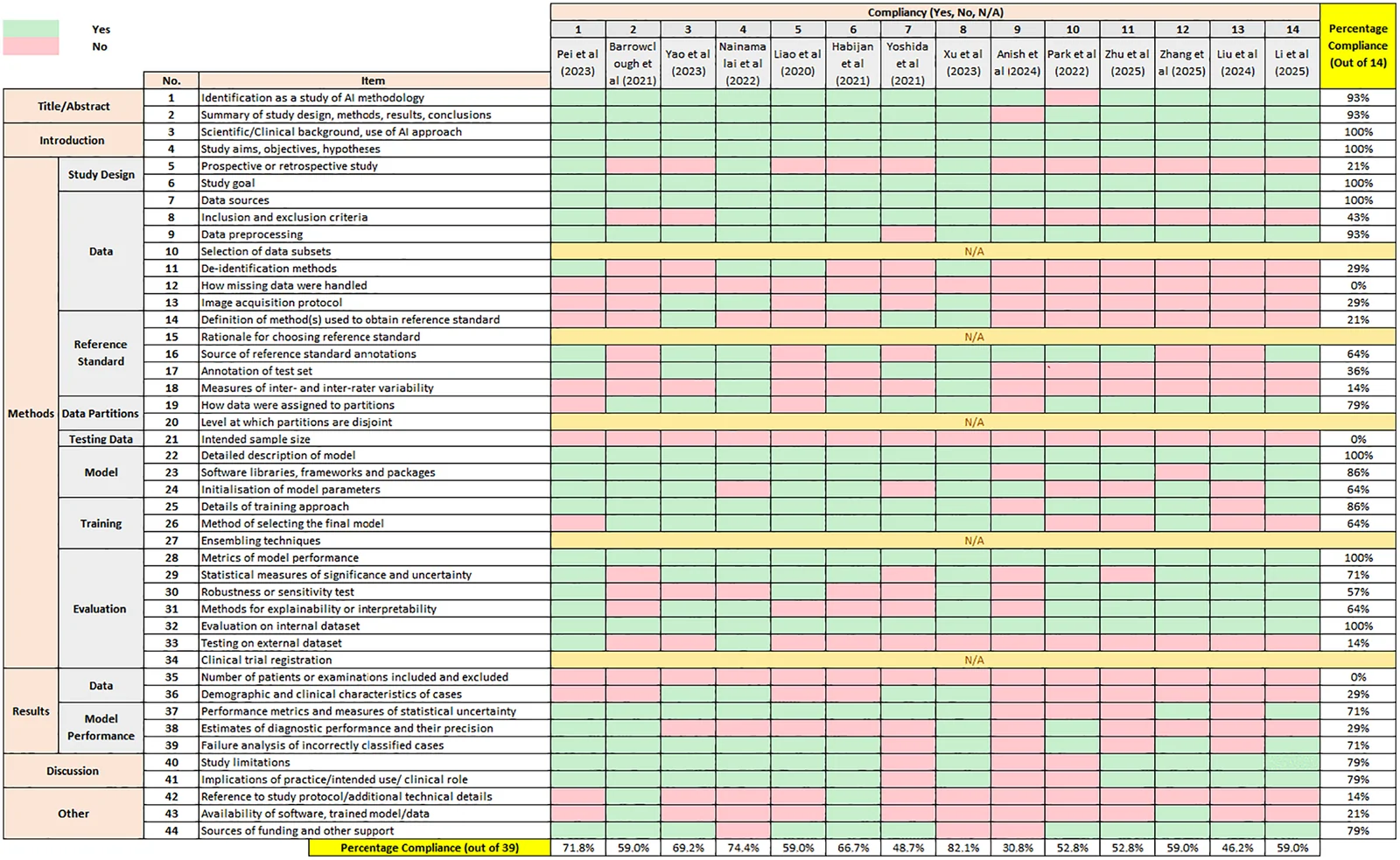
CLAIM checklist compliance against 44 criteria.

Reporting of dataset demographics and clinical characteristics were also limited with the inclusion and exclusion criteria rarely provided. Although data splitting and assignment to partitions were well documented in eleven studies [23,24,26,28,33,35,36], inconsistent use of terminology, such as the terms ‘validation’ and ‘testing’ used interchangeably compromises the reproducibility [45]. All 14 studies used patient-wise splitting, where all images from a given patient set are confined to a single partition (training, validation or testing). This approach is important to avoid information leakage where slices from one patient can appear in both training and testing sets, leading to biased performance estimates. The validation set is used during the model development process, to aid in fine-tuning of hyperparameters and prevent overfitting. Whereas the testing set is used after to evaluate the trained model’s performance on unseen images [46]. There should be no overlap between the training and testing datasets to prevent bias. Proper documentation and use of consistent terminology, especially when implementing niche or novel models, is essential in avoiding confusion and improving the reproducibility of studies.

### 4.4. Explainability and interpretability

The use of quantitative performance metrics alone is insufficient, explainable approaches such as using masked class activation maps (MCAM), Gradient-weighted class activation maps (Grad-CAM) and attention maps were used in two studies [28,34]. While heatmap approaches enhance post-hoc explainability by indicating important areas for model predictions, interpretability is not necessarily improved for radiologists who are accustomed to viewing grayscale images in dark environments [47]. Quantification of explainability and interpretability remains a challenge due to their subjective nature and misalignments with clinical workflows may hinder understanding rather than aid in diagnosis. As interpretability is based on individual user needs and levels of expertise, a user-centric approach should be taken to ensure outputs are clinically relevant and interpretable for the end user.

## 5. Study limitations

Our screening process did not incorporate a formal inter-reviewer agreement assessment such as Cohen’s Kappa, which may have introduced bias. This was because a consensus approach was employed where any disagreements between two reviewers were resolved through discussion until an agreement was reached. However, all screening decisions were reviewed independently by two investigators to minimise the risk of excluding relevant studies. This limitation should be taken into consideration when interpreting the findings. Additionally, this review was not prospectively registered on PROSPERO, however, the full predefined protocol is provided in supplementary materials S1 File.

## 6. Conclusion, future directions, and recommendations

Our work has revealed promising advancements in the field, such as a growing use of deep learning approaches for CT segmentation for paediatric CHD, development of novel methods and advanced hybrid approaches that demonstrate superior segmentation performance as well as robust clinical evaluation methods that pave the way towards clinical adoption. However, significant areas for improvement were also identified.

In more details, many studies critically under reported details on ground truth labelling, sample size determination, external dataset validation and integration of clinical expertise throughout the model development process. Furthermore, the limited availability of open-source datasets as well as significant dataset imbalance in which rarer complex CHD types are under-represented, collectively hinders advancement. In future, exploring generative AI techniques to augment training data with synthetic representations of rare CHD types may offer a promising strategy to mitigate class imbalance and improve model performance [48].

Addressing these gaps require adoption of standardised reporting protocols, rigorous experimental designs incorporating clinical domain knowledge and a multidisciplinary approach that actively involves clinicians not only in the ground truth labelling but validation and evaluation stages. Overall, through aligning deep learning model development with clinical needs and adopting a user-centric design, this can drive the development of robust, impactful, and clinically meaningful tools that enhance patient outcomes in congenital heart disease.

## Supporting information

S1 Fig Dataset imbalance for classification tasks. (DOCX)

S2 Fig Box and whisker plot showing the average Dice Similarity Coefficients for cardiac structures segmentation across 14 studies. (DOCX)

S1 Table Image pre-processing strategies across the 14 papers. (DOCX)

S2 Table Types of model frameworks mentioned across the 14 studies. (DOCX)

S3 Table Model architectures mentioned across the 14 studies. (DOCX)

S4 Table Table of Dice Similarity Coefficient values for cardiac structures segmentation across 14 studies. (DOCX)

S1 File Search strings, search strategy and data extraction strategy. (DOCX)

S2 File CLAIM Checklist scoring methodology. (DOCX)

S3 File Adapted PROBAST+AI risk of bias template designed for segmentation studies. (DOCX)

## References

[1] Su Z, Zou Z, Hay SI, Liu Y, Li S, Chen H, et al. Global, regional, and national time trends in mortality for congenital heart disease, 1990–2019: An age-period-cohort analysis for the Global Burden of Disease 2019 study. eClinicalMedicine. 2022;43:101249. 10.1016/j.eclinm.2021.101249 PMC8760503 35059612

[2] Bigras J-L. Cardiovascular Risk Factors in Patients With Congenital Heart Disease. Can J Cardiol. 2020;36(9):1458–66. doi: 10.1016/j.cjca.2020.06.013 32585329

[3] Pavlicek J, Klaskova E, Kapralova S, Palatova AM, Piegzova A, Spacek R, et al. Major heart defects: the diagnostic evaluations of first-year-olds. BMC Pediatr. 2021;21(1):528. doi: 10.1186/s12887-021-02997-2 34847867 PMC8630885

[4] Reddy VM. Low birth weight and very low birth weight neonates with congenital heart disease: timing of surgery, reasons for delaying or not delaying surgery. Semin Thorac Cardiovasc Surg Pediatr Card Surg Annu. 2013;16(1):13–20. doi: 10.1053/j.pcsu.2013.01.004 23561813

[5] Yang J, Veeraraghavan H, van Elmpt W, Dekker A, Gooding M, Sharp G. CT images with expert manual contours of thoracic cancer for benchmarking auto-segmentation accuracy. Med Phys. 2020;47(7):3250–5. doi: 10.1002/mp.14107 32128809 PMC8344378

[6] Xu Y, Quan R, Xu W, Huang Y, Chen X, Liu F. Advances in Medical Image Segmentation: A Comprehensive Review of Traditional, Deep Learning and Hybrid Approaches. Bioengineering (Basel). 2024;11(10):1034. doi: 10.3390/bioengineering11101034 39451409 PMC11505408

[7] Valverde I, Gomez G, Suarez-Mejias C, Hosseinpour A-R, Hazekamp M, Roest A, et al. 3D printed cardiovascular models for surgical planning in complex congenital heart diseases. Journal of Cardiovascular Magnetic Resonance. 2015;17:P196. doi: 10.1186/1532-429x-17-s1-p196

[8] Biglino G, Capelli C, Leaver L-K, Schievano S, Taylor AM, Wray J. Involving patients, families and medical staff in the evaluation of 3D printing models of congenital heart disease. Commun Med. 2015;12(2–3):157–69. doi: 10.1558/cam.28455 29048144

[9] Charlton TG, Hindocha S. Future Proofing the Clinical Oncology Workforce. ScienceDirect. 2023. 10.1016/j.clon.2023.02.002 36805130

[10] Bajwa J, Munir U, Nori A, Williams B. Artificial intelligence in healthcare: transforming the practice of medicine. Future Healthc J. 2021;8(2):e188–94. doi: 10.7861/fhj.2021-0095 34286183 PMC8285156

[11] Elhaddad M, Hamam S. AI-Driven Clinical Decision Support Systems: An Ongoing Pursuit of Potential. Cureus. 2024;16(4):e57728. doi: 10.7759/cureus.57728 38711724 PMC11073764

[12] Lin B, Tan Z, Mo Y, Yang X, Liu Y, Xu B. Intelligent oncology: The convergence of artificial intelligence and oncology. J Natl Cancer Cent. 2022;3(1):83–91. doi: 10.1016/j.jncc.2022.11.004 39036310 PMC11256531

[13] Shamshirband S, Fathi M, Dehzangi A, Chronopoulos AT, Alinejad-Rokny H. A review on deep learning approaches in healthcare systems: Taxonomies, challenges, and open issues. J Biomed Inform. 2021;113:103627. doi: 10.1016/j.jbi.2020.103627 33259944

[14] Obuchowicz R, Strzelecki M, Piórkowski A. Clinical Applications of Artificial Intelligence in Medical Imaging and Image Processing—A Review. Cancers. 2024;16(10):1870. 10.3390/cancers16101870 PMC11120567 38791949

[15] Chen W, Sun Q, Chen X, Xie G, Wu H, Xu C. Deep Learning Methods for Heart Sounds Classification: A Systematic Review. Entropy (Basel). 2021;23(6):667. doi: 10.3390/e23060667 34073201 PMC8229456

[16] de Siqueira VS, Borges MM, Furtado RG, Dourado CN, da Costa RM. Artificial intelligence applied to support medical decisions for the automatic analysis of echocardiogram images: a systematic review. Artificial Intelligence in Medicine. 2021;120:102165. 10.1016/j.artmed.2021.102165 34629153

[17] Jafari M, Shoeibi A, Khodatars M, Ghassemi N, Moridian P, Alizadehsani R, et al. Automated diagnosis of cardiovascular diseases from cardiac magnetic resonance imaging using deep learning models: A review. Comput Biol Med. 2023;160:106998. doi: 10.1016/j.compbiomed.2023.106998 37182422

[18] Chen C, Qin C, Qiu H, Tarroni G, Duan J, Bai W. Deep Learning for Cardiac Image Segmentation: A Review. Frontiers in Cardiovascular Medicine. 2020;7. 10.3389/fcvm.2020.00025 PMC7066212 32195270

[19] Alnasser TN, Abdulaal L, Maiter A, Sharkey M, Dwivedi K, Salehi M, et al. Advancements in cardiac structures segmentation: a comprehensive systematic review of deep learning in CT imaging. Front Cardiovasc Med. 2024;11:1323461. doi: 10.3389/fcvm.2024.1323461 38317865 PMC10839106

[20] Tejani AS, Tejani AS, Tejani AS, Tejani AS, Tejani AS, Tejani AS, et al. Checklist for Artificial Intelligence in Medical Imaging (CLAIM): 2024 Update. Radiology Artificial Intelligence. 2024;6(4). 10.1148/ryai.240300 PMC11304031 38809149

[21] Whiting PF, Rutjes AWS, Westwood ME, Mallett S, Deeks JJ, Reitsma JB, et al. QUADAS-2: a revised tool for the quality assessment of diagnostic accuracy studies. Ann Intern Med. 2011;155(8):529–36. doi: 10.7326/0003-4819-155-8-201110180-00009 22007046

[22] Moons KGM, Damen JAA, Kaul T, Hooft L, Andaur Navarro C, Dhiman P, et al. PROBAST+AI: an updated quality, risk of bias, and applicability assessment tool for prediction models using regression or artificial intelligence methods. BMJ. 2025;388:e082505. doi: 10.1136/bmj-2024-082505 40127903 PMC11931409

[23] Yao Z, Xie W, Zhang J, Yuan H, Huang M, Shi Y, et al. Graph matching and deep neural networks based whole heart and great vessel segmentation in congenital heart disease. Sci Rep. 2023;13(1):7558. doi: 10.1038/s41598-023-34013-1 37160940 PMC10169784

[24] Nainamalai V, Lippert M, Brun H, Elle OJ, Kumar RP. Local integration of deep learning for advanced visualization in congenital heart disease surgical planning. Intelligence-Based Medicine. 2022;6:100055. doi: 10.1016/j.ibmed.2022.100055

[25] Liao X, Qian Y, Chen Y, Xiong X, Wang Q, Heng P-A. MMTLNet: Multi-Modality Transfer Learning Network with adversarial training for 3D whole heart segmentation. Comput Med Imaging Graph. 2020;85:101785. doi: 10.1016/j.compmedimag.2020.101785 32898732

[26] Habijan M, Galić I, Leventić H, Romić K. Whole Heart Segmentation Using 3D FM-Pre-ResNet Encoder–Decoder Based Architecture with Variational Autoencoder Regularization. Applied Sciences. 2021;11(9):3912. doi: 10.3390/app11093912

[27] Anish TP, Joe Prathap PM. A novel approach for multi-CHD prediction using IW-LNEF-DJRNN model based on 3D CT images. Biomedical Signal Processing and Control. 2024;92:106074. doi: 10.1016/j.bspc.2024.106074

[28] Park S, Chung M. Cardiac segmentation on CT Images through shape-aware contour attentions. Comput Biol Med. 2022;147:105782. doi: 10.1016/j.compbiomed.2022.105782 35772330

[29] Barrowclough OJD, Muntingh G, Nainamalai V, Stangeby I. Binary segmentation of medical images using implicit spline representations and deep learning. Computer Aided Geometric Design. 2021;85:101972. doi: 10.1016/j.cagd.2021.101972

[30] Zhu Y, Li H, Cao B, Huang K, Liu J. A novel hybrid layer-based encoder-decoder framework for 3D segmentation in congenital heart disease. Sci Rep. 2025;15(1):11891. doi: 10.1038/s41598-025-96251-9 40195399 PMC11977193

[31] Zhang L, Hayashi Y, Oda M, Mori K. Double-mix pseudo-label framework: enhancing semi-supervised segmentation on category-imbalanced CT volumes. Int J Comput Assist Radiol Surg. 2025;20(5):991–1002. 10.1007/s11548-024-03281-1 PMC12055930 39932621

[32] Liu J, Zeng B, Chen X. Heart and great vessels segmentation in congenital heart disease via CNN and conditioned energy function postprocessing. Int J Comput Assist Radiol Surg. 2024;19(8):1597–605. doi: 10.1007/s11548-024-03182-3 38814529

[33] Li F, Li W, Peng Y, Shu Y. Whole heart segmentation based on 3D contour-guided multi-head attention network from CT and MRI images. IEEE Journal of Biomedical and Health Informatics. 2025;29(10):7459–72. 10.1109/JBHI.2025.3584074 40577307

[34] Pei Y, Shi G, Li J, Sun D, Wen C, Li J, et al. Anatomical attention-based prediction of postoperative pulmonary venous obstruction via CTA images. Comput Med Imaging Graph. 2023;103:102163. doi: 10.1016/j.compmedimag.2022.102163 36566530

[35] Yoshida A, Lee Y, Yoshimura N, Kuramoto T, Hasegawa A, Kanazawa T. Automated heart segmentation using U-Net in pediatric cardiac CT. Measurement: Sensors. 2021;18:100127. doi: 10.1016/j.measen.2021.100127

[36] Xu X, Jia Q, Yuan H, Qiu H, Dong Y, Xie W, et al. A clinically applicable AI system for diagnosis of congenital heart diseases based on computed tomography images. Med Image Anal. 2023;90:102953. doi: 10.1016/j.media.2023.102953 37734140

[37] Ronneberger O, Fischer P, Brox T. U-Net: Convolutional Networks for Biomedical Image Segmentation. Lecture Notes in Computer Science. Springer International Publishing. 2015. p. 234–41. doi: 10.1007/978-3-319-24574-4_28

[38] Liu J, Zeng B, Chen X. Heart and great vessels segmentation in congenital heart disease via CNN and conditioned energy function postprocessing. Int J Comput Assist Radiol Surg. 2024;19(8):1597–605. doi: 10.1007/s11548-024-03182-3 38814529

[39] Holt DB, El-Bokl A, Stromberg D, Taylor MD. Role of Artificial Intelligence in Congenital Heart Disease and Interventions. Journal of the Society for Cardiovascular Angiography & Interventions. 2025;4(3). 10.1016/j.jscai.2025.102567 PMC11993855 40230672

[40] Tonekaboni S, Joshi S, McCradden MD, Goldenberg A. What Clinicians Want: Contextualizing Explainable Machine Learning for Clinical End Use. proceedingsmlrpress. 2019:359–80.

[41] Sirocchi C, Sirocchi C, Sirocchi C. Medical-informed machine learning: integrating prior knowledge into medical decision systems. BMC Medical Informatics and Decision Making. 2024;24(S4). 10.1186/s12911-024-02582-4 PMC11212227 38943085

[42] Fouad S, Hakobyan L, Kavakli-Thorne M, Atkins S, Fratini A, Bhatia B. CHERIE: User-Centred Development of an XAI System for Chest Radiology through Co-Design. 2024.

[43] Wang Y, Han X, Li C, Luo L, Yin Q, Zhang J, et al. Impact of Gold-Standard Label Errors on Evaluating Performance of Deep Learning Models in Diabetic Retinopathy Screening: Nationwide Real-World Validation Study. J Med Internet Res. 2024;26:e52506. doi: 10.2196/52506 39141915 PMC11358665

[44] Faes L, Liu X, Wagner SK, Fu DJ, Balaskas K, Sim DA, et al. A Clinician’s Guide to Artificial Intelligence: How to Critically Appraise Machine Learning Studies. Transl Vis Sci Technol. 2020;9(2):7. doi: 10.1167/tvst.9.2.7 32704413 PMC7346877

[45] Kim DW, Jang HY, Ko Y, Son JH, Kim PH, Kim S-O, et al. Inconsistency in the use of the term “validation” in studies reporting the performance of deep learning algorithms in providing diagnosis from medical imaging. PLoS One. 2020;15(9):e0238908. doi: 10.1371/journal.pone.0238908 32915901 PMC7485764

[46] Duca AL. Understanding the Difference between Training, Test, and Validation Sets in Machine Learning. syntax-error. 2023.

[47] Ihongbe E, Fouad S, Mahmoud F, Rajasekaran A, Bhatia B. Evaluating explainable artificial intelligence (XAI) techniques in chest radiology imaging through a human-centered lens. PLOS ONE. 2024;19(10):e0308758. 10.1371/journal.pone.0308758 PMC11463756 39383147

[48] Kong F, Stocker S, Choi PS, Ma M, Ennis DB, Marsden AL. SDF4CHD: Generative modeling of cardiac anatomies with congenital heart defects. Medical Image Analysis. 2024;97. 10.1016/j.media.2024.103293 PMC11372630 39146700

[49] Zhuang X, Li L, Payer C, Štern D, Urschler M, Heinrich MP, et al. Evaluation of algorithms for Multi-Modality Whole Heart Segmentation: An open-access grand challenge. Med Image Anal. 2019;58:101537. doi: 10.1016/j.media.2019.101537 31446280 PMC6839613

[50] Xu X, Wang T, Shi Y, Yuan H, Jia Q, Huang M. Whole heart and great vessel segmentation in congenital heart disease using deep neural networks and graph matching. Lecture Notes in Computer Science. 2019. p. 477–85.

[51] Xu X, Wang T, Zhuang J, Yuan H, Huang M, Cen J, et al. ImageCHD: A 3D Computed Tomography Image Dataset for Classification of Congenital Heart Disease. Lecture Notes in Computer Science. Springer International Publishing. 2020. p. 77–87. doi: 10.1007/978-3-030-59719-1_8

