# Supplementary material for: Deep learning for cardiac CT segmentation for congenital heart disease: A systematic review
Source: PLoS One. 2026 Sep 29;21(9):e0357600. doi: 10.1371/journal.pone.0357600 (PMC13623130; doi:10.1371/journal.pone.0357600)
Supplement: Supplementary file 2 [file pone.0357600.s002.docx]

## **S2 Fig –** Box and whisker plot showing the average Dice Similarity Coefficients for cardiac structures segmentation across 14 studies
