# Supplementary material for: Deep learning for cardiac CT segmentation for congenital heart disease: A systematic review
Source: PLoS One. 2026 Sep 29;21(9):e0357600. doi: 10.1371/journal.pone.0357600 (PMC13623130; doi:10.1371/journal.pone.0357600)
Supplement: Supplementary file 5 [file pone.0357600.s005.docx]

## **S3 Table -** Model architectures mentioned across the 14 studies

| **Model Architecture** | **Description** | **Study** |
| --- | --- | --- |
| **UNet**  (37) | The UNet is composed of an encoder-decoder structure. The unique design includes skip connections which connects layers from the encoder with corresponding layers in the decoder. The connecting paths concatenates features from the encoder with the decoder, this allows subsequent upsampling convolutional layer to operate over both features, capturing both spatial and semantic information. | Liao et al. (2020)  Pei et al. (2023)  Yoshida et al. (2021)  Barrowclough et at (2021)  Yao et al. (2023)  Xu et al. (2023)  Liu et al. (2024)  Li et al. (2025) |
| **CNN**   | Convolutional Neural Networks (CNNs) are primarily linear in structure, consisting of a sequential arrangement of multiple layers, which include convolutional layers, max-pooling layers, and fully connected layers. This arrangement allows for extraction of hierarchical features from input images. | Park et al. (2022)  Nainamalai et al. (2022)  Zhang et al. (2025) |
| **RNN**   | Recurrent Neural Networks (RNNs) process information sequentially, element by element, with loop structures allowing for information to be retained. The general structure consists of three main components, the input layer, recurrent layer, and the output layer, x0, x1, x2 represent input values used to predict the next value in the sequence. | Anish et al. (2024) |
| **GAN**   | The structure of Generative Adversarial Networks (GANs) consists of two parts: the generator and the discriminator. The generator is responsible for curating synthetic data that resembles real data, and discriminator acts as a classifier that differentiates between real data from the training set and fake data created by the generator. This adversarial process generates highly realistic output images. | Liao et al. (2020) |
| **Advanced Hybrid Deep Learning Models**    (25) | Advanced hybrid deep learning models integrate multiple deep learning architectures to enhance performance for complex tasks by leveraging each of their strengths. Examples include Res-U-Net a combination of UNet and Residual networks (ResNet).  The hybrid model proposed by Liao et al (2020) combined UNet, GAN and CNN-based architectures. | Pei et al (2023)  Liao et al (2020)  Habijan et al (2021)  Zhu et al. (2025)  Li et al. (2025) |
