# Supplementary material for: Deep learning for cardiac CT segmentation for congenital heart disease: A systematic review
Source: PLoS One. 2026 Sep 29;21(9):e0357600. doi: 10.1371/journal.pone.0357600 (PMC13623130; doi:10.1371/journal.pone.0357600)
Supplement: Supplementary file 7 [file pone.0357600.s007.docx]

## **S1 File** – Search strings, search strategy and data extraction strategy

A comprehensive search was conducted across six databases: PubMed, IEEE Xplore, MDPI, Science Direct, Web of Science and Scopus. Search date was filtered from 2004 to 2025 (31^st^ October). No database level filters (language restrictions, publication type etc.) were applied during database searches to maximise retrieval. Records were subsequently filtered using the reference manager Endnote X9.3.3, this included exclusion of non-journal publications and any records that did not meet the inclusion criteria.

**Web of science**

((ALL = Congenital heart disease) AND (ALL = CT) AND (ALL = Cardiac) AND (ALL=Segmentation) AND (ALL = Artificial Intelligence)) OR ((ALL = Congenital heart disease) AND (ALL = CT) AND (ALL = Cardiac) AND (ALL=Segmentation) AND (ALL = Machine learning)) OR ((ALL = Congenital heart disease) AND (ALL = CT) AND (ALL = Cardiac) AND (ALL=Segmentation) AND (ALL = Deep learning))

**PubMed**

(Congenital heart disease AND CT AND Cardiac AND Segmentation AND Artificial Intelligence) OR (Congenital heart disease AND CT AND Cardiac AND Segmentation AND Machine learning) OR (Congenital heart disease AND CT AND Cardiac AND Segmentation AND Deep learning)

**IEEE XPLORE**

("Congenital heart disease" AND "CT" AND "Cardiac" AND "Segmentation" AND "Artificial Intelligence") OR ("congenital heart disease" AND "CT" AND "Cardiac" AND "Segmentation" AND "Machine learning") OR ("congenital heart disease" AND "CT" AND "Cardiac" AND "Segmentation" AND "Deep Learning")

**MDPI**

No string allowed so advanced search was used

**Science Direct**

("Congenital heart disease" AND "CT" AND "Cardiac" AND "Segmentation" AND "Artificial Intelligence")

("Congenital heart disease" AND "CT" AND "Cardiac" AND "Segmentation" AND "Machine Learning")

("Congenital heart disease" AND "CT" AND "Cardiac" AND "Segmentation" AND "Deep Learning”)

**Scopus**

Filters used: ‘Limited to Article’

((ALL = Congenital heart disease) AND (ALL = CT) AND (ALL = Cardiac) AND (ALL=Segmentation) AND (ALL = Artificial Intelligence)) OR ((ALL = Congenital heart disease) AND (ALL = CT) AND (ALL = Cardiac) AND (ALL=Segmentation) AND (ALL = Machine learning)) OR ((ALL = Congenital heart disease) AND (ALL = CT) AND (ALL = Cardiac) AND (ALL=Segmentation) AND (ALL = Deep learning))

Data Extraction strategy

Data extraction was performed dual independently by two investigators (SY and AF). A structured extraction table was developed prior to commencing the extraction. Extracted features were recorded in the table, this is presented in Figure 2 of the manuscript. The extracted features included: Study characteristics: Study Title, Name of 1^st^ Author, Country of 1^st^ Author, Publication Year. Dataset characteristics: Imaging Modality, CT Machine Type, Imaging Protocol, Source of Dataset, Patient Demographics, Dataset Size, Train, Test and Validation split, Cardiac structures segmented, CHD Type. Research design characteristics: Annotation software, ground truth labeller, ground truth validator. Any discrepancies identified during the verification process were resolved through discussion and consensus between the two investigators. Study authors were not contacted regarding missing or unclear information as we wanted to accurately document reporting practices. In cases where methodological details were not reported in the publication or supplementary materials, this was documented as “Not mentioned”.
