# Supplementary material for: Deep learning for cardiac CT segmentation for congenital heart disease: A systematic review
Source: PLoS One. 2026 Sep 29;21(9):e0357600. doi: 10.1371/journal.pone.0357600 (PMC13623130; doi:10.1371/journal.pone.0357600)
Supplement: Supplementary file 8 [file pone.0357600.s008.docx]

## **S2 File** – CLAIM Checklist scoring methodology

The most up to date CLAIM 2024 checklist was used to assess reporting quality of each of the studies. Five of the CLAIM criteria were excluded as they were deemed inapplicable or irrelevant for the purposes of comparison and for ease of calculation overall CLAIM compliance. Specifically, these included:

Criteria 10) Selection of subsets.

Justification: This criterion describes selecting subsets of raw extracted data during preprocessing (e.g. selective cropping or image exclusion). None of the included studies reported investigator driven selection of subsets beyond standard preprocessing steps applied uniformly across the dataset, therefore this was deemed not applicable.

Criteria 15) Rationale for choosing reference standard.

Justification: This criterion is intended to assess studies where multiple competing reference standards are available. In included medical imaging studies, there reference standard was consistently manual annotation performed by clinical experts or trained annotators which represents the gold standard in this domain. No alternative reference standards were considered across these studies, so this criterion was deemed not applicable.

Criteria 20) Level at which partitions are disjoint.

Justification: This criterion evaluates whether data was split at patient or image level to prevent information leakage. All the included studies reported data partitioning at patient-level which is the consensus and standard for medical imaging studies. As no studies employed image-level partitioning, this criterion was considered not applicable.

Criteria 27) Ensembling Techniques.

Justification: The presence or absence of ensembling does not represent a reporting or methodological weakness, as single-model approaches are common and appropriate in medical image segmentation studies. For example, scoring this criterion as “No” for studies that did not use ensembles would incorrectly penalise them for modelling choice, therefore this criterion was considered not applicable.

Criteria 34) Clinical Trial registration.

Justification: The reviewed studies were retrospective, methodological segmentation studies that did not require clinical trials involving patient recruitment. Clinical trial registration is therefore not applicable.
