# Supplementary material for: Deep learning for cardiac CT segmentation for congenital heart disease: A systematic review
Source: PLoS One. 2026 Sep 29;21(9):e0357600. doi: 10.1371/journal.pone.0357600 (PMC13623130; doi:10.1371/journal.pone.0357600)
Supplement: Supplementary file 9 [file pone.0357600.s009.docx]

## **S3 File** - Adapted PROBAST+AI risk of bias template designed for segmentation studies

**PROBAST+AI Adapted for Medical Imaging Segmentation Studies**

*Quality, Risk of Bias, and Applicability Assessment Tool*

*Adapted from: Moons KGM, Damen JAA, Kaul T, et al. PROBAST+AI: an updated quality, risk of bias, and applicability assessment tool for prediction models using regression or artificial intelligence methods. BMJ 2025;388:e082505*

This adaptation modifies the original PROBAST+AI tool specifically for deep learning medical imaging segmentation studies, where the task is pixel/voxel-level classification rather than patient-level outcome prediction.

**Key Adaptations for Segmentation Studies**

| **Original PROBAST+AI Concept** | **Segmentation Adaptation** |
| --- | --- |
| Predictors | Input images and preprocessing pipeline |
| Outcome | Reference standard segmentation (ground truth) |
| Calibration | Not directly applicable |
| Discrimination (AUC, c-index) | Dice coefficient, Intersection over Union (IoU) |
| Additional metrics | Hausdorff distance, surface distance, volumetric accuracy |
| Events per variable | Cases/images per model complexity (less clearly defined for DL) |

**Step 1: Define the Review Question**

*Complete once per systematic review or assessment to define the intended purpose.*

| **Item** | **Description** |
| --- | --- |
| **Population** | Define the target patient population and imaging characteristics (e.g., paediatric patients with congenital heart disease undergoing cardiac CT) |
| **Index Model(s)** | Define the segmentation models being assessed (e.g., nnU-Net models for cardiac substructure segmentation) |
| **Comparator Model(s)** | Define comparison models if applicable (e.g., U-Net baseline, atlas-based methods, manual segmentation) |
| **Target Structures** | Define the anatomical structures to be segmented (e.g., left ventricle, right ventricle, myocardium, great vessels) |
| **Imaging Modality** | Define the imaging modality and acquisition parameters (e.g., contrast-enhanced cardiac CT, MRI sequences) |
| **Setting and Intended Use** | Define the clinical setting and intended application (e.g., preoperative planning, treatment response monitoring, research) |

**Step 2: Classify the Type of Segmentation Study**

*Complete once for each study being assessed. Different signalling questions apply for different study types.*

| **Study Type** | **Description** | **Sections to Complete** |
| --- | --- | --- |
| Development only | Segmentation model development without external evaluation of performance | Model Development |
| Evaluation only | External validation of existing segmentation model(s) on new data | Model Evaluation |
| Combination | Model development combined with evaluation (internal validation, cross-validation, or external validation) | Model Development AND Model Evaluation |

**Classification for this study:**

[ ] Development only    [ ] Evaluation only    [ ] Combination

**Step 3: Quality and Applicability Assessment**

| **Publication Reference** |  |
| --- | --- |
| **Model(s) of Interest** |  |
| **Target Structures** |  |

**MODEL DEVELOPMENT**

*Rate each domain for quality concerns (low/high/unclear). Signalling questions rated as Y (yes), PY (probably yes), PN (probably no), N (no), NI (no information), or NA (not applicable). All questions phrased so that 'yes' or ‘probably yes’ indicates low concern for quality. All questions rated ‘no’ or ‘probably no’ flags the potential for sincere concerns regarding the quality of that domain. You will need to use your judgement to determine whether the entire domain should be rated as ‘High’, ‘Low’, or ‘Unclear’ overall.*

**DOMAIN 1: Participants and Imaging Data Sources**

**A. Quality**

*Describe the imaging data sources, patient population, scanner information, acquisition protocols, and inclusion/exclusion criteria:*

| 1.1 Were appropriate imaging data sources used (institution, scanner type, acquisition dates)? | *Y/ PY/ PN/ N/ NI* |
| --- | --- |
| 1.2 Was an appropriate study design used (prospective collection, established registry, or well-documented retrospective database)? | *Y/ PY/ PN/ N/ NI* |
| 1.3 Did in/exclusions result in a dataset representative of the target population, including appropriate variation in image quality, scanner vendors, and patient characteristics? | *Y/ PY/ PN/ N/ NI* |
| 1.4 Was multi-center data included, or was single-center limitation acknowledged? | *Y/ PY/ PN/ N/ NI* |
| **Quality concern for participant and data source selection** | *LOW / HIGH / UNCLEAR* |

*Rationale for quality rating:*

**B. Applicability**

*Describe the imaging data sources, patient characteristics, clinical setting, scanner details, and date range:*

| **Concern that the imaging data, patient population, or acquisition parameters do not match the review question or intended clinical use** | *LOW / HIGH / UNCLEAR* |
| --- | --- |

*Rationale for applicability rating:*

**DOMAIN 2: Input Images and Preprocessing**

**A. Quality**

*Describe the input image characteristics (modality, resolution, dimensions), preprocessing steps (resampling, normalisation, cropping, artifact correction), and data augmentation methods:*

| 2.1 Were imaging acquisition parameters consistent or appropriately documented across all cases? | *Y/ PY/ PN/ N/ NI* |
| --- | --- |
| 2.2 Was image preprocessing (resampling, intensity normalisation, cropping, artifact correction) applied consistently across all cases? | *Y/ PY/ PN/ N/ NI* |
| 2.3 Was the preprocessing pipeline fully described and reproducible? | *Y/ PY/ PN/ N/ NI* |
| 2.4 Were data augmentation methods appropriate and clearly reported? | *Y/ PY/ PN/ N/ NI* |
| **Quality concern for input images and preprocessing** | *LOW / HIGH / UNCLEAR* |

*Rationale for quality rating:*

**B. Applicability**

| **Concern that the imaging modality, acquisition parameters, or preprocessing do not match the review question or intended clinical use** | *LOW / HIGH / UNCLEAR* |
| --- | --- |

*Rationale for applicability rating:*

**DOMAIN 3: Reference Standard Segmentation (Ground Truth)**

**A. Quality**

*Describe the reference standard segmentation method (manual annotation, semi-automatic, consensus), annotator qualifications, annotation protocol, number of annotators, and inter-rater reliability if assessed:*

| 3.1 Was the reference standard segmentation created using an appropriate method (expert manual annotation, consensus protocol, or validated semi-automatic method)? | *Y/ PY/ PN/ N/ NI* |
| --- | --- |
| 3.2 Were annotator qualifications and experience level appropriate and reported? | *Y/ PY/ PN/ N/ NI* |
| 3.3 Was the reference standard generated using consistent annotation protocols across all cases? | *Y/ PY/ PN/ N/ NI* |
| 3.4 Was inter-rater and/or intra-rater reliability assessed and reported? | *Y/ PY/ PN/ N/ NI* |
| 3.5 Were the number of annotators and consensus method (if any) clearly described? | *Y/ PY/ PN/ N/ NI* |
| 3.6 Were annotation guidelines/protocols documented? | *Y/ PY/ PN/ N/ NI* |
| **Quality concern for reference standard segmentation** | *LOW / HIGH / UNCLEAR* |

*Rationale for quality rating:*

**B. Applicability**

*List the structures segmented and their definitions:*

| **Concern that the target structures, their definitions, or annotation method do not match the review question or intended clinical use** | *LOW / HIGH / UNCLEAR* |
| --- | --- |

*Rationale for applicability rating:*

**DOMAIN 4: Analysis (Model Development)**

**Quality**

*Describe the dataset size (number of patients/cases, number of images/volumes), model architecture, training procedure, and methods to address overfitting:*

*Describe missing data handling and any post-processing applied to segmentation outputs:*

| 4.1 Was there evidence that the sample size (number of cases/images) was adequate for the model complexity? | *Y/ PY/ PN/ N/ NI* |
| --- | --- |
| 4.2 Were image intensity values and spatial information handled appropriately (normalisation, resolution standardisation)? | *Y/ PY/ PN/ N/ NI* |
| 4.3 Were cases with missing structures, poor image quality, or incomplete annotations handled appropriately? | *Y/ PY/ PN/ N/ NI* |
| 4.4 If methods to address class imbalance (foreground/background or between structures) were used, were they appropriate? | *Y/ PY/ PN/ N/ NI* |
| 4.5 Were appropriate methods used to address overfitting (regularisation, dropout, early stopping, appropriate architecture)? | *Y/ PY/ PN/ N/ NI* |
| 4.6 Was data splitting performed at the patient/subject level (not image/slice level)? | *Y/ PY/ PN/ N/ NI* |
| 4.7 Was any post-processing clearly described and applied consistently? | *Y/ PY/ PN/ N/ NI* |
| **Quality concern for the analysis** | *LOW / HIGH / UNCLEAR* |

*Rationale for quality rating:*

**MODEL EVALUATION**

*Rate each domain for risk of bias (low/high/unclear). If investigators only performed performance evaluation on Training set, responses from the Model Development section can be copied for Domains 1-3. Domain 4 should be evaluated separately for each validation type: T = Training, I = Internal, E = External.*

**DOMAIN 1: Participants and Imaging Data Sources**

**A. Risk of Bias**

*Describe the evaluation data sources, patient population, scanner information, and inclusion/exclusion criteria:*

| 1.1 Were appropriate imaging data sources used? | *Y/ PY/ PN/ N/ NI* |
| --- | --- |
| 1.2 Was an appropriate study design used for evaluation? | *Y/ PY/ PN/ N/ NI* |
| 1.3 Did in/exclusions result in a representative evaluation dataset? | *Y/ PY/ PN/ N/ NI* |
| **Risk of bias from participant and data source selection** | *LOW / HIGH / UNCLEAR* |

*Rationale for risk of bias rating:*

**B. Applicability**

| **Concern that evaluation data do not match the review question or intended use** | *LOW / HIGH / UNCLEAR* |
| --- | --- |

*Rationale for applicability rating:*

**DOMAIN 2: Input Images and Preprocessing**

**A. Risk of Bias**

*Describe the input image characteristics and preprocessing applied in the evaluation data:*

| 2.1 Were imaging parameters consistent or appropriately documented in evaluation data? | *Y/ PY/ PN/ N/ NI* |
| --- | --- |
| 2.2 Was preprocessing applied consistently in evaluation data? | *Y/ PY/ PN/ N/ NI* |
| 2.3 Was preprocessing pipeline the same as used during model development? | *Y/ PY/ PN/ N/ NI* |
| **Risk of bias from input images and preprocessing** | *LOW / HIGH / UNCLEAR* |

*Rationale for risk of bias rating:*

**B. Applicability**

| **Concern that imaging or preprocessing in evaluation do not match the review question** | *LOW / HIGH / UNCLEAR* |
| --- | --- |

*Rationale for applicability rating:*

**DOMAIN 3: Reference Standard Segmentation**

**A. Risk of Bias**

*Describe the reference standard used in evaluation, annotator qualifications, and whether it differs from development:*

| 3.1 Was the reference standard in evaluation created using an appropriate method (expert manual annotation, consensus protocol or validated semi-automatic method)? | *Y/ PY/ PN/ N/ NI* |
| --- | --- |
| 3.2 Was the reference standard generated consistently across evaluation cases? | *Y/ PY/ PN/ N/ NI* |
| 3.3 Were the same structures segmented as in model development? | *Y/ PY/ PN/ N/ NI* |
| **Risk of bias from reference standard segmentation** | *LOW / HIGH / UNCLEAR* |

*Rationale for risk of bias rating:*

**B. Applicability**

| **Concern that the reference standard in evaluation does not match the review question** | *LOW / HIGH / UNCLEAR* |
| --- | --- |

*Rationale for applicability rating:*

**DOMAIN 4: Analysis (Model Evaluation)**

**Risk of Bias**

*Describe the evaluation dataset size, validation approach (cross-validation, held-out test set, external), and performance metrics reported:*

*Validation type: T = Training, I = Internal validation, E = External validation. Shaded cells indicate question does not apply to that validation type.*

| **Signalling Question** |  | |
| --- | --- | --- |
| 4.1 Was model evaluation based on only Training performance? | Y/PY/PN/N/NI  If ‘Y/PY’ Risk = High | |
| **Signalling Question** | **Internal** | **External** |
| 4.2 Was there evidence that the evaluation sample size was adequate? | Y/PY/PN/N/NI | Y/PY/PN/N/NI |
| 4.3 Were cases with missing data or poor quality handled appropriately? | Y/PY/PN/N/NI | Y/PY/PN/N/NI |
| 4.4 If class imbalance methods were used in development, was evaluation done without imbalance correction? | Y/PY/PN/N/NI | Y/PY/PN/N/NI |
| 4.5 Was data splitting performed at patient level and was data leakage avoided? | Y/PY/PN/N/NI | Y/PY/PN/N/NI |
| 4.6 If resampling/cross-validation was used, were all development steps replicated in each fold? | Y/PY/PN/N/NI | Y/PY/PN/N/NI |
| 4.7 Were multiple appropriate segmentation metrics reported (overlap AND boundary)? | Y/PY/PN/N/NI | Y/PY/PN/N/NI |
| 4.8 Was performance reported separately for each structure (for multi-structure segmentation)? | Y/PY/PN/N/NI | Y/PY/PN/N/NI |
| 4.9 Were metrics reported with appropriate uncertainty (CI, SD)? | Y/PY/PN/N/NI | Y/PY/PN/N/NI |
| 4.10 Was performance stratified by relevant subgroups (disease severity, image quality, scanner)? | Y/PY/PN/N/NI | Y/PY/PN/N/NI |

| **Risk of bias from the analysis** | *LOW / HIGH / UNCLEAR* |
| --- | --- |

*Rationale for risk of bias rating:*

**Step 4: Overall Judgement**

**Model Development**

| **Overall Quality Concern** | **Criteria** |
| --- | --- |
| Low concern | All four domains rated low concern |
| High concern | At least one domain rated high concern |
| Unclear concern | At least one domain unclear, none high |

| **Overall quality concern (Model development)** | *LOW / HIGH / UNCLEAR* |
| --- | --- |
| **Overall applicability concern (Model development)** | *LOW / HIGH / UNCLEAR* |

*Summary of quality and applicability concerns (development):*

**Model Evaluation**

| **Overall Risk of Bias** | **Criteria** |
| --- | --- |
| Low risk | All four domains rated low risk |
| High risk | At least one domain rated high risk |
| Unclear risk | At least one domain unclear, none high |

| **Overall risk of bias (Model evaluation)** | *LOW / HIGH / UNCLEAR* |
| --- | --- |
| **Overall applicability concern (Model evaluation)** | *LOW / HIGH / UNCLEAR* |

*Summary of risk of bias and applicability concerns (evaluation):*
